# Competitive Pricing Using Model-Based Bandits

**DOI:** 10.1007/s10614-024-10816-w

**Published:** 2025-02-04

**Authors:** Lukasz Sliwinski, Tanut Treetanthiploet, David Siska, Lukasz Szpruch

**Affiliations:** 1 Maxwell Institute for Mathematical Sciences, School of Mathematics, University of Edinburgh, Edinburgh, EH9 3FD UK; 2 The Institute for Fundamental Study, Naresuan University, Phitsanulok, 65000 Thailand; 3 Quantum Technology Foundation (Thailand), Bangkok, 10110 Thailand; 4 Alan Turing Institute, 96 Euston Road, London, NW1 2DB UK

**Keywords:** Algorithmic pricing, Multi-armed bandits, Pricing game

## Abstract

The use of learning algorithms for automatic price adjustments in markets is on the rise. However, these algorithms often assume that reward distributions for actions are uncorrelated and stationary, a condition that does not hold in competitive pricing environments. In this paper, we introduce a pricing environment, find conditions under which a unique Nash equilibrium exists and verify the assumptions numerically. Then, we propose a bandit algorithm that approximates the structure of the environment and extend it to accommodate non-stationary settings. We perform numerical tests in both stationary and competitive pricing environments, analysing the potential benefits and drawbacks of incorporating the structure of the environment within learning algorithms. While modelling the stationary environment improves the algorithm’s performance in a stationary setting, it does not offer an advantage in pricing competitions between non-stationary learning agents.

## Introduction

In electronic markets, an increasing number of decisions are made by automated algorithms. When these algorithms adapt using arriving new information, their performance might be difficult to predict. There exist a myriad of approaches for optimal control and learning within environments that are stationary with respect to time. In stationary case, when the environment is unknown, some of these algorithms come with theoretical guarantees that their performance will converge toward the optimal behaviour. In finite-dimensional learning problems, where the number of actions and states is finite, a well-known example of such an algorithm is the Q-learning algorithm introduced by Watkins and Dayan (1992). In infinite-dimensional problems, there exist results for convergence of learning algorithms in special cases. For example, Kerimkulov et al. (2023) prove the exponential convergence of mirror descent for Markov Decision Problems with possibly infinite-dimensional state and action spaces. In practice, however, environments are often non-stationary.

In this paper, we focus on multi-agent setting which can be regarded as non-stationary environment from the viewpoint of a single agent. The environment, as perceived by an individual agent, includes the dynamics and the strategies of competing agents. Consequently, if the behaviour of competing agents exhibits non-stationarity, the environment itself becomes non-stationary. This scenario is evident in settings where multiple agents learn simultaneously within a shared environment. The internal state and the strategies of the agents change with time and thus the environment, as seen by any single agent, also evolves with time.

One of such multi-agent environments is any financial market with a few dealers/ providers of a product, e.g. liquidity markets, goods markets, web advertising and markets of particular services such as insurance. If one dealer decreases the quoted prices, all other dealers have to follow, which in turn affects the initial dealer. Thus, the environment, as perceived by a single dealer, changes.

It is tempting to apply the algorithms derived for stationary or simple non-stationary settings to multi-agent environment, in hope that the algorithms will provide satisfactory performance. In this paper, we will study the application of bandit algorithms to a pricing problem, but we will vary the complexity of the algorithms and their assumptions about the environment. As a result, we will study whether improvements to bandit algorithms can yield good performance in multi-agent problems.

Recent interest in the topic of algorithmic pricing was sparked by numerous cases where the use of algorithms has led to market inefficiencies and supra-competitive pricing. Solon (2011) describes a case where algorithmic pricing on Amazon led to the listing price of a book reaching 24 million dollars. Although this has been a clear error that can be easily detected, this is not true in general. In Assad et al. (2020), the authors inspected real-world data and showed that the introduction of automated pricing at gas stations is linked to higher gas prices with an increase ranging from 9 to 28%, levels that cannot be detected by retail customers. The topic of tacit collusion was further investigated by numerical studies. Calvano et al. (2020) presented a numerical study in which the competition between two Q-learning algorithms converged to collusive equilibria. That is, equilibria in which both agents quoted prices higher than one-stage Nash equilibrium prices and punished any deviations from those prices. Consequently, the authors concluded that the algorithms have learned to collude without explicit communication. While other studies such as den Boer et al. (2022) contested the conclusion that the algorithms in fact learned to collude, it cannot be disputed that algorithmic pricing can lead to higher prices.

In this paper, we focus on a simple pricing environment in which companies compete to try to find the optimal one-stage pricing strategy, where the agents aim to maximise the expected reward at each step. We derive the environment using the bottom-up approach. We first focus on the micro-structure of a single auction and then, by taking expectation over the sources of randomness, derive the expected rewards given the pricing strategies of all agents. Next, we study the Nash equilibria within the environment. First, we prove the existence of at least one Nash equilibrium. Then, we formulate sufficient conditions for the existence of a unique Nash equilibrium. We verify numerically that the assumptions hold in the region of interest.

Then, we perform numerical experiments of the pricing competition between two agents that employ simple bandit algorithms to learn and compete within the environment. For that purpose, we formulate the bandit model of the environment which approximates the derived pricing environment. In addition to that, we vary the decision rule that agents use to sample the next action. We analyse the results of the simulations, focusing on the performance of agents and the pricing strategies that the agents converge to.

Our study builds on the work by Cartea et al. (2022). In their paper, the authors investigate an over-the-counter liquidity market. They show the conditions for existence of the Nash equilibrium and then perform numerical experiments with four types of bandit algorithms. The authors conclude that some of the algorithms can indeed converge towards supra-competitive pricing. In their work, most of the used bandit algorithms assumed stationarity of the environment, which, as we explained, is not true in multi-agent settings.

The paramount questions that appear in Cartea et al. (2022) are: “What are the types of equilibria that algorithmic pricing systems converge to?” and “How does the choice of competing algorithms dictate which equilibrium is achieved?”.

This question was further investigated in Cartea et al. (2022a) and Cartea et al. (2022c). In Cartea et al. (2022a), the authors develop a tool that transcribes discrete dynamics of learning schemes into a smooth dynamical system and then prove the asymptotic convergence between the two in the limit of decreasing time step. Thus, given the learning algorithms, the derived dynamics can be used to simulate the asymptotic evolution of the system. In Cartea et al. (2022c), the authors use similar stochastic approximation tools to show that a smooth fictitious play converges to any stable Nash equilibrium with non-zero probability.

In addition, the work presented here builds on the model-based approach developed in Cohen and Treetanthiploet (2021), where the authors tackled the dynamic pricing problem using the Asymptotic Randomised Control (ARC) algorithm. The algorithm leveraged prior information about the structure of the information to guide optimal exploration and exploitation. Our work similarly employs the prior information about the structure of the pricing environment but joins this information with standard exploration/exploitation rules, which enables us to factor and analyse the effect of the prior information on the performance of the bandits.

The ARC algorithm Cohen and Treetanthiploet (2020), along with other influential studies of bandit problems such as Even-Dar et al. (2006), Auer and Ortner (2010) and Ryzhov et al. (2012) provides a perspective into optimal solution to the general stationary multi-armed problem which is essential when extending the problem to multi-agent settings.

The non-stationarity modification of the algorithms used in this work was inspired by Garivier and Moulines (2008) where the authors present two approaches for non-stationarity and derive the upper and lower bounds for regret growth. The important paper by Besbes et al. (2015) provides a generalised framework for measuring non-stationarity within the bandit settings.

For a valuable insight into model-based and model-free reinforcement learning in the context of insurance pricing, refer to Treetanthiploet et al. (2023). The authors employed a model-based approach to establish an initial pricing strategy, followed by the application of model-free updates to improve the strategy in an online setting.

Lastly, our work is closely related to the problem of optimal bidding, in which advertisers bid in real-time first price auctions for ad impressions from ad publishers. Yuan et al. (2014) provides a survey of the ad real-time-bidding while Ou et al. (2023) offers an overview of the research on algorithms for the bid optimisation.

The current practice for automated pricing often involves offline optimisation based on the historical data or model of the environment (Ou et al., 2023; de Larrard, 2016). When online learning is introduced, it often involves a periodic re-learning - repeating offline optimisation with new data. As such, these strategies erroneously assume that the agents within the environments have fixed strategies. This assumption is flawed in dynamic and competitive environments where the strategies of agents are continually adapting.

Our work, by contrast, focuses on pricing competitions between purely non-stationary, continuously learning algorithms, where the strategy is not influenced by long-running histories of observations. We analyse how these types of algorithms perform in pricing competitions and the equilibria to which they converge.

A secondary objective of this study is to determine whether having a parametric model of the environment can enhance an agent’s performance in a pricing competition. In theory, a parametric model can increase sample efficiency and accelerate learning. Consequently, if an agent learns faster than its competitors, it should be able to quote better prices and achieve higher profits.

To summarise, the main contributions of this work are:

- Demonstrating the existence and uniqueness of a Nash equilibrium for the one-stage pricing game, given certain assumptions on the payoff function.
- Establishing the convergence of best response play to the unique Nash equilibrium, under the assumptions on the payoff function.
- Introducing a bandit model of the pricing environment.
- Conducting numerical analysis to verify whether incorporating prior structural information into the bandit algorithms improves the performance of the agent.
- Simulating the competition between purely non-stationary learning algorithms and analysis of the equilibria to which the algorithms converge.

The numerical experiments were implemented using Python. The code necessary to reproduce the obtained results can be accessed through a Github repository. For further details, please refer to the Code Availability Statement.

## Pricing Environment

There are *N* agents, which represent companies that compete for items. At each time step, there is a new item. Each agent proposes a price, denoted as $P_t^i$, where *i* denotes the agent $i \in \{1,2,...,N\}$ and $t \in {\mathbb {N}}$ denotes the time step. The item will be sold to the *i*-th agent if the *i*-th agent’s price is the lowest and it is smaller than some reservation price, denoted as $P_t^C$. When the agent manages to secure, it will receive a reward $R^i_t$ equal to the price of the offer minus the *i*-th agent’s true value of the item $V_t^i$.

We write for the reward at step *t* for *i*-th agent, given all the prices and values

1 $$\begin{aligned} R_t^i = \mathbbm {1}_{P^i_t< \min _{j\ne i} P_t^j }\mathbbm {1}_{P^i_t< P^V_t } \Big (P_t^i - V_t^i\Big ). \end{aligned}$$

We make assumptions on each agent’s item valuation and the reservation price.

1. The value vector $V_t$ follows a normal distribution centred around some mean value *M* with variance $\omega ^2$ and correlation $\rho$. Denoting ${\textbf{1}}$ as a column vector, we have:
  2 $$\begin{aligned} V_t \sim {\mathcal {N}}({\textbf{1}}M, \Omega ),\quad \text { where }\quad \Omega _{ij} = \left\{ \begin{aligned}&\omega ^2 \ \,\qquad \text {if } i=j, \\&\rho \omega ^2 \qquad \text {if } i\ne j. \end{aligned}\right. \end{aligned}$$
2. The reservation price $P_t^C$ is normally distributed with mean $M+S^C$ and variance $\tau ^2$, where $S^C$, the reservation margin, is fixed, so that
  3 $$\begin{aligned} P_t^C \sim {\mathcal {N}}(M+S^C, \tau ^2) \end{aligned}$$

The agent chooses a margin $S_t^i$ and then quotes the price

4 $$\begin{aligned} P^i_t = V^i_t + S_t^i. \end{aligned}$$

The expected reward for an agent, given other agents’ margins is

5 $$\begin{aligned} {\mathbb {E}}\left[ R^i_t | S_t^1, S_t^2, ..., S_t^N\right] = {\mathbb {E}}\left[ S_t^i\mathbbm {1}_{S_t^i + V_t^i< \min _{j\ne i} (S_t^j + V_t^j)} \mathbbm {1}_{S_t^i + V_t^i < P_t^C } \right] . \end{aligned}$$

The pricing environment described above can be applied in various scenarios. One potential application is in online insurance pricing, where customers from different segments visit price comparison websites to obtain quotes. The moral hazard associated with each customer, as estimated by an insurance company, can be modelled as the item valuation $V_t$. Another application is in real-time bidding for ad impressions, where the value of a newly created impression, as estimated by marketers, can similarly be modelled as $V_t$.

### Expected Rewards

We derive analytical expressions for the expected rewards and offer acceptance probabilities over a single time step given all the agents’ margins $S_t = \{S_t^1, S_t^2,..., S_t^N\}$. Since the randomness is stationary, let us drop the time subscript.

#### Lemma 1

Let $\varphi$ denote the probability density function of the standard normal variable ${\mathcal {N}}(0,1)$ and let $\Phi$ denote the associated cumulative distribution function. Given all agents’ margins $S = \{S^1, S^2,..., S^N\}$, the expected reward of *i*-th agent is given by

6 $$\begin{aligned} &  {\mathbb {E}}\left[ R^{i}\Big | S \right] = {\mathbb {E}}\left[ S^i\mathbbm {1}_{S^i + V^i< \min _{j\ne i} (S^j + V^j)} \mathbbm {1}_{S^i + V^i < P^C} \Big | S \right] \nonumber \\ &  \quad = S^i \int _{\mathbb {R}} \Phi \left( -\frac{S^{i} - S^{C} +\omega \sqrt{1-\rho }x }{\sqrt{\tau ^2 + \rho \omega ^2}}\right) \prod _{j\ne i} \Phi \left( -\frac{S^i - S^{j} +\omega \sqrt{1-\rho }x}{\omega \sqrt{1-\rho }}\right) \varphi (x)dx. \end{aligned}$$

The offer acceptance probability is

7 $$\begin{aligned} \begin{aligned}&{\mathbb {E}}\left[ \mathbbm {1}_{S^i + V^i< \min _{j\ne i} (S^j + V^j)} \mathbbm {1}_{S^i + V^i < P^C} \Big | S \right] \\&\quad = \int _{\mathbb {R}} \Phi \left( -\frac{S^{i} - S^{C} +\omega \sqrt{1-\rho }x }{\sqrt{\tau ^2 + \rho \omega ^2}}\right) \prod _{j\ne i} \Phi \left( -\frac{S^{i} - S^{j} +\omega \sqrt{1-\rho }x}{\omega \sqrt{1-\rho }}\right) \varphi (x) dx. \end{aligned} \end{aligned}$$

For the proof of Lemma 1, see Appendix A.1.

**Fig. 1:**
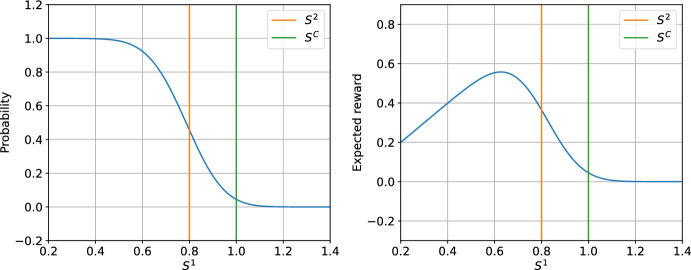
Offer acceptance probability (left panel) and expected reward (right panel) for the agent with index 1 as a function of $S^{1}$ given other parameters fixed: $S^{2}=0.8$, $S^C=1.$, $\omega = 0.15$, $\tau = 0.13$, $\rho = 0.5$

Figure 1 presents the probability of accepting an offer and the expected reward as a function of the agent’s margin for a sample two-agent setup.

### Agent Objectives, Strategies and Nash Equilibrium

In this setting, the agent will focus on maximisation of a step reward. The *i*-th agent’s action is the chosen margin $S^i$ with the corresponding action space ${\mathcal {S}} \subset {\mathbb {R}}$. Given all agents’ actions (the quoted margins), we can calculate the payoff for each agent using the expected reward function (6). The action spaces and the payoff functions induce a game between agents, where each agent has to maximise the expected payoff. Each agent adopts a strategy, which represents a distribution over the action space ${\mathcal {S}}$. Let us denote the strategy of the *i*-th agent as $\pi ^i$ and the corresponding space of probability measures as ${\mathcal {P}}$.

We now define the concepts of pure strategy, mixed strategy, Nash equilibrium, and best response.

#### Definition 1

A pure strategy is a strategy that dictates that the agent commits to a single action (margin) with probability 1.

#### Definition 2

A mixed strategy is a strategy that specifies a probability distribution over the action space from which the agent samples the action (margin).

Note that the action and strategy space are the same for all agents. The expected payoff for the agent is then defined as

8 $$\begin{aligned} J_i(\pi ^i, \pi ^{(-i)}) = {\mathbb {E}}_{S^{(-i)} \sim \pi ^{(-i)}} \bigg [ {\mathbb {E}}_{S^i \sim \pi ^i} \Big [{\mathbb {E}}[R^i | S^i, S^{(-i)}] \Big ]\bigg ], \end{aligned}$$

where $$\pi ^{(-i)}$$ denotes $$[\pi ^1, \pi ^2,..., \pi ^{i-1}, \pi ^{i+1},...,\pi ^N]$$.

#### Definition 3

A set of strategies $\pi ^* = \{\pi ^{1*}, \pi ^{2*},..., \pi ^{N*}\}$ is called a Nash equilibrium if no agent can improve their payoff function given that the strategies of the other agents are kept constant. Given that for all *i*, $\pi ^i \in {\mathbb {R}}$, we can write the condition as

9 $$\begin{aligned} \forall _{i\in \{1,2,...,N\}} \ \forall _{\pi ^{i} \in {\mathcal {P}}}\quad J_i\left( \pi ^{i}, \pi ^{(-i*)}\right) \le J_i\left( \pi ^{i*}, \pi ^{(-i*)}\right) . \end{aligned}$$

Furthermore, a Nash equilibrium is called “pure” if all the strategies in $\pi ^*$ are pure. Otherwise, the Nash equilibrium is called “mixed”.

Finally, we introduce the concept of the best response.

#### Definition 4

Best response mapping $B_i:{\mathcal {P}}^{N-1} \rightarrow \sigma ({\mathcal {P}})$ takes the vector of the other agents’ strategies $\pi ^{(-i)}$ and returns a set of strategies of the *i*-th player which maximise the payoff of the *i*-th agent $J_i$

10 $$\begin{aligned} B_i\left( \pi ^{(-i)}\right) = \arg \max _{\pi ^i \in {\mathcal {P}}} J_i\Big (\pi ^i, \pi ^{(-i)}\Big ). \end{aligned}$$

Clearly, for a Nash equilibrium $\pi ^*$, $\pi ^{i*} \in B_i\left( \pi ^{(-i*)}\right)$. Each agent’s equilibrium strategy is optimal with respect to the equilibrium strategies of other agents.

We now formulate a theorem reqarding existence of a Nash equilibrium for the pricing game.

#### Theorem 1

Let the agents’ action space ${\mathcal {S}}$ be convex and compact. Then, for a one-stage pricing game with payoff functions given by (8), there exists a Nash equilibrium.

#### Proof

The theorem is a result of the Glicksberg theorem (Glicksberg, 1952). Glicksberg’s theorem requires that the number of agents is finite, the action space is convex compact and the payoff functions are continuous. $\square$

The additional assumption that the action space ${\mathcal {S}}$ is compact and convex is grounded in practice. It implies that there are both lower and upper bounds on the margins quoted by the agents.

The above theorem gives us the existence of one or more Nash equilibria in either pure or mixed strategies. In the following two sections, we develop reasonable assumptions about the expected reward function (6). Under these assumptions, we show that there exists a unique Nash equilibrium and that all of the corresponding strategies are pure strategies.

### Assumptions on the Expected Reward Function

Looking at the expression for the expected reward (6), we note that it is a continuous and differentiable function of *S*. Now, we make assumptions on the expected reward function ${\mathbb {E}}[R^i|S]$, consistent with Fig. 1:

#### Assumption 1

For all $$i \in \{1,2,...,N \}$$:

(i) *Existence of a unique maximizer.* For any
  11 $$\begin{aligned} S^{(-i)} = [S^1, S^2, ..., S^{i-1}, S^{i+1},...,S^N] \in {\mathbb {R}}^{N-1}, \end{aligned}$$
  there exists a unique positive maximiser ${\mathbb {E}}[R^i|S^i,S^{(-i)}]$. Consequently, we define the best response function $B_i: {\mathbb {R}}^{N-1} \rightarrow {\mathbb {R}}$:
  12 $$\begin{aligned} B_i\left( S^{(-i)}\right) = \arg \max _{S^i \in {\mathbb {R}}} {\mathbb {E}}\left[ R^i|S^i,S^{(-i)}\right] . \end{aligned}$$
  We further assume that $B_i$ is differentiable with respect to its arguments.
(ii) *Monotonicity of the maximiser with respect to other margins* $S^{j}$. For all $j\ne i$
  13 $$\begin{aligned} \frac{\partial B_i}{\partial S^{j}} >0. \end{aligned}$$
(iii) *Linear growth of the maximiser with respect to other margins* $S^{j}$.
  14 $$\begin{aligned} \sum _{j\ne i} \frac{\partial B_i}{\partial S^{j}} \le \beta , \end{aligned}$$
  for some $$\beta \in (0,1)$$.

Assumption 1-(ii) indicates that if any other agent increases their margin, the optimal margin of the *i*-th agent will strictly increase. Assumption 1-(iii) asserts that if all other agents increase their margins by a fixed amount $d>0$, the maximiser will increase by at most $\beta d$. If all other agents decrease their margins by a fixed amount $d>0$, the maximiser will decrease by at most $\beta d$. For heuristic motivation and discussion of Assumption 1-(iii), see Appendix D.

**Fig. 2:**
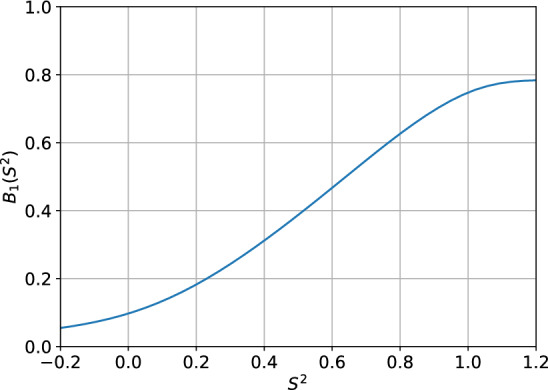
Best response function for agent with index 1 as a function of the other agent’s margin $S^{2}$ given other parameters fixed: $S^C=1.$, $\omega = 0.15$, $\tau = 0.13$, $\rho = 0.5$

Figure 2 presents the best response function for a two-player setup. The plot agrees with the assumptions - the best response function is monotonically increasing with a slope less than 1. Furthermore, the assumptions were tested in a three-player setup and none of the assumptions was violated. Appendix E contains the results of the performed numerical tests.

Furthermore, Appendix C presents an alternative pricing environment used in the economic literature (Calvano et al., 2020) that satisfies Assumption 1.

### Uniqueness of Nash Equilibrium Under Assumption 1

Under Assumption 1, we can define a transform $B(S): {\mathbb {R}}^N \rightarrow {\mathbb {R}}^N$ such that

15 $$\begin{aligned} B(S) = \left[ B_1\big (S^{(-1)}\big ), B_2\big (S^{(-2)}\big ), ...,B_N\big (S^{(-N)}\big ) \right] . \end{aligned}$$

For each vector of margins *S*, the transform *B* returns a vector of margins $S'$ where each margin $S'^i$ is the best response to the opponent margins in *S*.

#### Lemma 2

For $x \in {\mathbb {R}}^N$, let $|x|_\infty := \max _{i \in \{1,2,...,N\}} |x_i|$. Under Assumption 1, the transform *B* is a contraction. For any $S_a, S_b \in {\mathbb {R}}^N$:

16 $$\begin{aligned} \big |B(S_a) - B(S_b) \big |_\infty \le \beta \big |S_a - S_b \big |_\infty \end{aligned}$$

where the value $\beta$ is given by Assumption 1.

The lemma is a consequence of the sublinearity of the maximiser in Assumption 1(iii). For the detailed proof, see Appendix A.2.

The space of actions with the supremum norm $({\mathbb {R}}^N, |\cdot |_\infty )$ is a complete metric space and *B* is a contraction on that space, and thus the Banach fixed point theorem implies the existence of a unique fixed point $S^* = B(S^*)$. By inspection, the unique fixed point defines a unique pure Nash equilibrium for the pricing game. This is summarised in the following theorem.

#### Theorem 2

Let Assumption 1 hold. The best response transform *B* has a unique fixed point in ${\mathbb {R}}^N$, denoted as $S^*$. Consequently, the single-stage pricing game has exactly one Nash equilibrium in pure strategies where for each *i*, *i*-th agent chooses the margin $S^{i*}$.

Next, we formulate a theorem regarding the Nash equilibrium in mixed strategies.

#### Theorem 3

Let Assumption 1 hold. The single-stage pricing game has no mixed Nash equilibria except for the unique pure Nash equilibrium specified in Theorem 2.

Lastly, we formulate a theorem about the convergence of the best-response play.

#### Theorem 4

Let Assumption 1 hold. In an iterated pricing game with mixed strategies, where each agent plays any best response to the agents’ mixed strategies from the previous round, the played strategies converge in distribution to the unique pure Nash equilibrium.

For the proof of Theorems 3 and 4, see Appendix B. The proof is mostly technical - the main idea is to use the contraction property of the transform *B* to prove that the supports of the mixed policies will contract due to application of the best response play.

Convergence to the Nash equilibrium requires information about the other agents’ strategies and the knowledge of the environment to compute the best response, which is not the case in practice. Nonetheless, the characteristics of the environment suggest that the agents who optimise for the best response are likely to converge towards the Nash equilibrium.

## Pricing as Multi-Armed Bandit Problem

The previous sections discussed the structure of the environment and the potential characteristics of that environment. In the following, we focus on algorithms that can be implemented in practice to learn a strategy (margin) that will maximise the profit. Specifically, we will treat the pricing problem as a multi-armed bandit problem and we will employ Bayesian perspective as in Kaufmann et al. (2012) and Scott (2010).

In a general multi-armed bandit (MAB) problem, at each time step $t \in {\mathbb {N}}$, the agent chooses an action $a_t$ from one of the *K* available actions that results in an observation $y_t$ and a reward $r_t:=r(y_t)$. The observation is sampled from the distribution $\Pi _{\theta }(a_t)$ where $\theta$ is a parameter and $a_t$ is the chosen action. Although the agent does not know the true value of $\theta$, it knows the functional form of the distribution $\Pi _\theta$ and has prior information on $\theta$. Using that prior information and the history of observations, the agent updates its belief about the parameters $\theta$. Agent strategy $\pi$ is a decision rule that at each step *t* outputs a probability over *K* actions given the information collected up to time *t*. Consequently, through sampling, this provides an action $a_t \sim \pi$. The agent’s goal is to maximise the expected accumulated reward over *T* steps:

17 $$\begin{aligned} {\mathbb {E}}\left[ \sum _{t=1}^T r_t | a_t\right] . \end{aligned}$$

In the above description, we have further generalised the problem so that in addition to the reward $r(y_t)$, the agent can receive additional information in the observation vector $y_t$.

It is important to note that in a general multi-armed bandit problem, there is only one agent which interacts with a stationary environment. This is different in our case. Even if the pricing environment is stationary, there can be multiple competing agents. Other agents do not have fixed strategies, they also learn and adjust their margins, rendering the problem non-stationary, as seen from the point of a single learning agent.

This does not mean that algorithms for solving MABs cannot be applied - the main idea is to treat other agents as a part of the environment and model it accordingly. One approach is to include other agents within the model and thus explicitly compute the probabilities of actions from other competing agents. However, this necessitates additional information about the number of agents and their behavior, which is typically unavailable.

Our approach is thus different, we first develop stationary models which approximate the environment when the other agents are fixed. Then, we adapt these models to account for the potential non-stationarity of the environment, allowing for changes in their parameters over time.

### Bandit Algorithms

Two key components of Bayesian MAB algorithms are:

1. An assumed form of the distribution $\Pi _{\theta }$, an assumption regarding the stationarity of the parameters $\theta _t$, prior information over the parameters and a corresponding (possibly approximate) posterior update based on the history of observations,
2. A decision rule - a function which takes all the available information and outputs a probability distribution over the available actions.

The two aspects listed above can be chosen independently of each other. We will refer to the form of the observation distribution with the reward function as the bandit model of the environment. The method of computing the probability distribution for the action will be referred to as the bandit decision rule.

We further illustrate the underlying structure of the bandit algorithms using a meta-algorithm presented as Algorithm 1.

**Algorithm 1:**
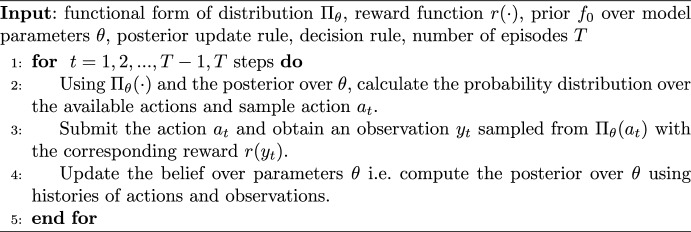
Meta bandit algorithm

Even for the classic bandit problem, where the model assumes that the distributions of rewards for each arm are independent, there is no analytical solution for the optimal strategy. Thus, bandit algorithm decision rules often rely on regret bounds (e.g. UCB from Auer and Ortner (2010)) or heuristic rules (e.g. Eps-Greedy, see Section 3.4 of Scott (2010)).

The next sections focus on bandit models of the environment that will be applied to the pricing problem. Afterwards, the paper will introduce decision rules that will be employed in the numerical experiments later.

### Stationary Classical Model

One of the most widely used bandit models of the environment is the classical model. The classical model assumes that for each of the *K* available actions, denoted as $\{a^1,a^2,...,a^K\}$, there is a stationary model parameter $\{\theta ^1,\theta ^2,...,\theta ^K\}$. The observation is the reward, which can be expressed as $r(y_t)=y_t$. After choosing the *i* -th action at time step *t*, $a_t=a^i$, the reward/observation for the agent is assumed to be sampled from a normal distribution with mean $\theta ^i$ and variance $\delta$, where $\delta$ is fixed and assumed within the prior information. That is, the main assumption of the model is

18 $$\begin{aligned} r_t\ |\ (a_t\!=\!a^i) \sim {\mathcal {N}}(\theta ^i, \delta ). \end{aligned}$$

The model starts with a given prior on each of the parameters and the fixed parameter $\delta >0$. In the classical model, each parameter has a normal uncorrelated prior

19 $$\begin{aligned} \theta _i \sim {\mathcal {N}}(m_0^i, d_0^i), \end{aligned}$$

where for each *i*

20 $$\begin{aligned} m_0^i =0 \qquad \qquad (d_0^i)^{-1} = 0. \end{aligned}$$

Additionally, throughout this paper, the reward variance $\delta$ will be set to 1, which will match the magnitude of the prospective reward.

As $(d_0^i)^{-1} = 0$ implies that $d_0^i =\infty$, care must be taken to ensure that the rest of the calculations are well defined. This is true in the case of the classical model - the update calculations are defined using the inverse of $d_0$. Due care is also needed when specifying the valuation of actions, and such instances will be noted and explained.

At each time step, the agent updates the posterior. The posterior at each time step is normal

21 $$\begin{aligned} \theta _i \sim {\mathcal {N}}(m^i_t, d^i_t). \end{aligned}$$

If agent chooses action $a_i$ and observes the reward $r^i_t$, the posterior for parameter $\theta _i$ is updated as following

22 $$\begin{aligned} d^i_t&= \frac{1}{\delta ^{-1} + (d^i_{t-1})^{-1}} = d^i_{t-1}\left( 1 - \frac{d^i_{t-1}}{d^i_{t-1} + \delta }\right) , \end{aligned}$$

23 $$\begin{aligned} m^i_t&= d^i_t \left( \delta ^{-1}r_t + (d^i_{t-1})^{-1}m_{t-1}^i\right) . \end{aligned}$$

If the action *i* is not taken then the posterior parameters are unchanged. For the proof of the update equations (22) and (23), see Appendix F.1.

### Stationary Logistic Model

In the previous section, the classical bandit model employed the assumption that the rewards for each action are independent. Consequently, the true mean reward for action *i* - the average reward obtained after sampling action *i* infinitely many times - is independent of the rewards collected for actions $j\ne i$. Therefore, the only method to learn about the true mean reward for action *i* is to sample action *i*. This is not the case for the pricing environment. Lemma 1 shows that the rewards are not independent, since the expected reward is a function of the margin given by Equation (6). Therefore, collecting the reward corresponding to the margin $s^i$ provides us with knowledge about the true mean reward corresponding to other margins. If a bandit model was able to tractably process that information, it would have an advantage over the classical model.

Looking at the graphs in Fig. 1, we see that the reward curve and the probability curve have a distinct shape. The probability curve is smoothly decreasing while the expected reward curve increases linearly until a global maximum and then decays towards zero. The stationary logistic model aims to capture some of this structure of the pricing environment. Specifically, the prior information of the model will contain the approximate relationship between the margin and the probability of the offer acceptance. In the actual model of the environment with which the agents will interact, this relation is expressed by the formula (7). For the logistic model, this relation will be modelled using logistic regression. The probability plot in Fig. 1 suggests that, for fixed opponent margins, logistic regression could effectively approximate the actual probability.

At each time step, the agent submits a margin $s_t$ (sampled according to the strategy) which, for the purpose of the model, translates to an action vector $a_t$ such that

24 $$\begin{aligned} a_t = [1, s_t]^T. \end{aligned}$$

The observation random variable $Y_t \in \{0,1\}$ models rejection/acceptance and is assumed to follow a Bernoulli distribution, where the probability parameter is given by a logistic function

25 $$\begin{aligned} Y_t \sim \text {Bernoulli}(p_t),\text { where }\quad p_t = \zeta \left( \theta ^T a_t\right) := \frac{1}{1+ \exp \left( -\theta ^T a_t\right) }, \end{aligned}$$

where $\theta \in {\mathbb {R}}^2$ is the model parameter vector. This is the key assumption of the model that originates from the inspection of Fig. 1. After sampling $Y_t$ to obtain $y_t$, the reward for the agent becomes

26 $$\begin{aligned} r_t = y_t s_t. \end{aligned}$$

The key advantage of the logistic model over the classical model is that it assumes that the distributions of the rewards for each action are correlated. Thus, if the agent chooses an action with a margin $s_k$ and receives an observation, they will gain information not only about the reward distribution corresponding to the submitted margin $s_k$, but also about the parameters of the model and consequently also about the reward distributions for the other available actions/margins. Thus, the agent should learn more efficiently and find the optimal actions faster.

The logistic model does not require a discrete action set. However, for purpose of comparison with the classic model, the set of available margins will be discrete, and unless specified otherwise, it will be equal to the set of margins available within the classic model.

#### Logistic Model Error

**Fig. 3:**
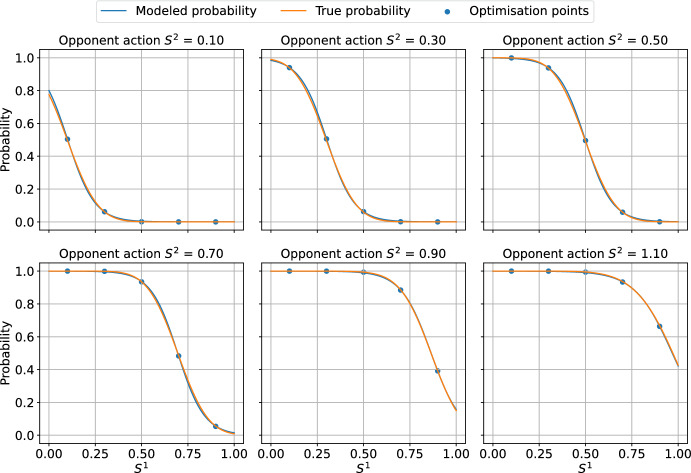
Approximations of the true probability curves using logistic regression. The regression was performed using the collocation points corresponding to the available actions

To assess the accuracy of the logistic model, we performed an error analysis for the environment configurations presented in the following sections. For the environment parameters, see Sect. 4. For each opponent action, we have optimised the logistic model to minimise the mean squared error between the modelled probability and the true probability, evaluated at the available agent actions. Figure 3 presents the true vs. learnt probability curves, together with the points for which the model was optimised. Table 1 lists the computed errors for each agent action for each logistic regression specified by the opponent action. The errors indicate that the logistic model approximates the environment well - the errors at the collocation points, that is available actions, are of the order $10^{-3}$ and the supremum errors are of order $10^{-2}$. This conclusion is reinforced by the plots in Fig. 3. We can conclude that for every opponent action, there exists a value of $\theta$ for which the logistic regression provides a reliable approximation of the expected reward function.

**Fig. 4:**
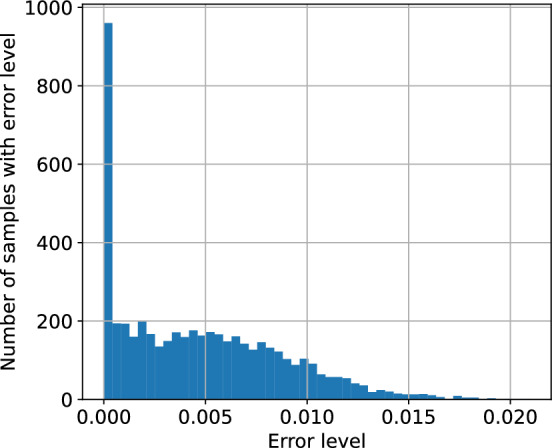
Histogram of errors collected over all samples and all collocation points

**Fig. 5:**
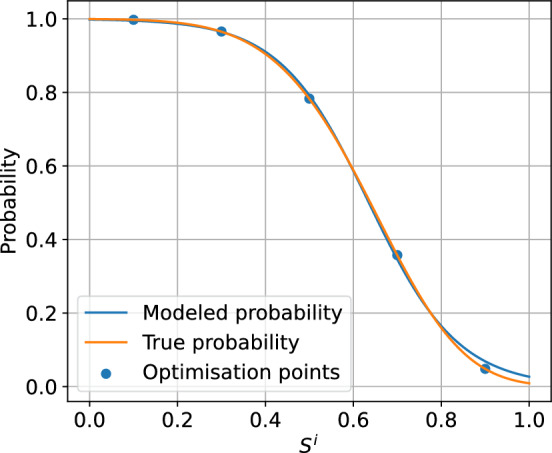
Regression of logistic model for the highest error. The opponent margins for the worst case were [0.5, 1.1, 1.1] while the cost standard deviation $\omega =0.36$ and $\rho =0.03$

The above error analysis was extended to include 3 opponents (total of 4 agents in the environment) and variable environment parameters, specifically the standard deviation of the costs $\omega$ and the correlation of the costs $\rho$. To overcome the computational complexity, the error analysis was performed using random sampling. The opponent margins were uniformly sampled from a set $\{0.1,0.3,0.5,0.7,0.9,1.1\}$. The standard deviation $\omega$ was sampled uniformly from a range [0.05, 0.55] and the correlation factor was sampled from a range [0, 1]. The number of samples was 2000. For each sample, the logistic model was first optimised using the available actions as collocation points. Then, the regressed model was used to calculate the errors at the collocation points. Figure 4 presents a histogram of errors collected across all samples at all collocation points. The plot indicates that most of the errors are of the order $10^{-3}$, but there are a significant number of errors of the order $10^{-2}$ with a maximum error of around 0.02. Figure 5 presents the regression plot for the worst-case error, that is, the plot where this maximum error occurs. As the caption suggests, for the worst case, the opponent margins were relatively high ([0.5, 1.1, 1.1]), the cost standard deviation was high ($\omega =0.36$) and the correlation was close to 0 ($\rho =0.03$).

**Table 1:** Errors in the approximation of the probability function

Opponent action	Agent action					
	0.1	0.3	0.5	0.7	0.9	Supremum over [0., 1.]
0.1	5.0e$-$05	5.1e$-$04	3.4e$-$03	3.2e$-$04	2.2e$-$05	1.8e$-$02
0.3	6.5e$-$04	2.4e$-$04	1.1e$-$04	3.5e$-$03	3.0e$-$04	2.4e$-$02
0.5	3.2e$-$03	1.4e$-$03	4.1e$-$04	4.0e$-$04	3.4e$-$03	2.4e$-$02
0.7	2.9e$-$04	3.2e$-$03	2.5e$-$03	1.0e$-$03	2.4e$-$03	1.6e$-$02
0.9	7.8e$-$05	6.9e$-$04	3.5e$-$03	7.7e$-$04	1.7e$-$04	1.1e$-$02
1.1	1.7e$-$04	1.1e$-$03	3.6e$-$03	1.1e$-$03	1.6e$-$04	6.0e$-$03

Opponent action signifies the fixed margin quoted by the opponent and corresponds to a single logistic model regression

#### Prior on the Model Parameters and Posterior Update

Having defined the logistic model, we need a method to process the knowledge about the model parameters that will subsequently allow the algorithm to use the collected information to optimise future decisions. The information processing method should satisfy the following requirements: it has to be tractable and consistent. Tractability implies that the posterior can be easily computed, while consistency requires that as the model receives more observations, the posterior mean converges to the true parameters, and the variance converges to 0.

Taking into account the above, we will approximate the posterior at each time step using a normal distribution. Specifically, at each time step, we will compute the exact posterior using the Bayes rule. Then, we will apply the second-order approximation to the non-linear terms of the log posterior around a specified mean *m*. The resulting approximate posterior will form a normal distribution with a new mean and covariance matrix.

This approximate posterior meets the tractability requirement: at each step the model knowledge is parametrised solely by a vector of means and a covariance matrix. Furthermore, approximating the posterior with a normal distribution is motivated by the Bernstein–von Mises theorem (Nickl, 2012; Van der Vaart, 2000) which states that under some mild assumptions the posterior converges to a normal distribution. Consequently, this suggests that with the appropriate choice for the mean *m* used for the second-order approximation, the update method could be consistent.

Thus, at time $t=0$, we start with a normal prior

27 $$\begin{aligned} \theta \sim {\mathcal {N}}(m_0, d_0), \end{aligned}$$

where $d_0$ is necessarily large to encode the uncertainty about the exact value of the parameters.

After a sequence of actions up to time *t*, $\{a_1, a_2,...,a_t\}$, and the corresponding observations $\{y_1, y_2,...,y_t\}$, the posterior is approximated as normal distribution

28 $$\begin{aligned} \theta \sim {\mathcal {N}}(m_t,d_t). \end{aligned}$$

Denoting the mean for second-order approximation as *m*, the posterior parameters $m_t$ and $d_t$ are computed as follows:

29 $$\begin{aligned} d_{t}&= \left( d_0^{-1} + S_t \right) ^{-1} \end{aligned}$$

30 $$\begin{aligned} m_{t}&= d_{t} \left( d_0^{-1} \mu _0 + S_t m + \sum _{k=1}^{t}(y_{k} - \zeta ( m^T a_k)) a_k\right) \end{aligned}$$

31 $$\begin{aligned} \text {with} \quad S_t :&= \sum _{k=1}^{t} \zeta (m^T a_k)(1-\zeta ( m^T a_k)) a_ka_k^T. \end{aligned}$$

For the derivation of the approximate posterior, see Appendix F.2.

The choice of the mean *m* for the second-order expansion is important, and a wrong choice might cause the approximation to diverge. For example, the mean can be calculated as maximum a posteriori point $m_{MAP}$ such that

32 $$\begin{aligned} m_{MAP} = \text {argmax}_\theta \log \pi (Y_1=y_1,Y_2=y_2,....,Y_t=y_t, \theta | m_0,d_0), \end{aligned}$$

where $\pi$ denotes the exact posterior. This choice is motivated by the Laplace approximation (MacKay, 2003) and the consistency of the maximum likelihood estimator for logistic regression (Rashid, 2009) (under some regularity conditions). While having good convergence characteristics, it requires solving an optimisation problem at each update of the posterior.

An alternative method employed in this work utilises the parameter mean calculated in the previous step $m_{t-1}$ so that

33 $$\begin{aligned} d_{t}&= \left( d_0^{-1} + S_t \right) ^{-1} \end{aligned}$$

34 $$\begin{aligned} m_{t}&= d_{t} \left( d_0^{-1} \mu _0 + S_t m_{t-1} + \sum _{k=0}^{t-1}(y_{k} - \zeta ( m_{t-1}^T a_k)) a_k\right) \end{aligned}$$

35 $$\begin{aligned} \text {with} \quad S_t :&= \sum _{k=1}^{t} \zeta (m_{t-1}^T a_k)(1-\zeta ( m_{t-1}^T a_k)) a_ka_k^T. \end{aligned}$$

This approximation is much faster but may lead to instabilities at initial steps, where a limited number of observations can skew the mean towards values with very high magnitudes. This would then influence the subsequently calculated posterior means. To overcome this instability, the posterior update included a stabilisation function. If the computed mean or the norm of the covariance matrix fell outside the prespecified bounds, then the new mean would be computed using the update (34) with $m_{t-1}$ set to $m_0$.

### Non-Stationary Models - Agents Assuming Non-Stationary Environments

Non-stationary models include the agent’s prior belief that the parameters of the observation distribution can change with time. In this paper, the non-stationary models will be obtained from stationary models by applying the sliding-window (SW) approach introduced in Garivier and Moulines (2008). This modification dictates that the posterior is calculated using only a specified number of recent observations. The parameter controlling the sliding window will be denoted as $w \in {\mathbb {N}}$ and will be referred to as the window size. The lower the value of *w*, the faster the model will forget the past experience.

For the classical model presented in Sect. 3.2, the new posterior will still be a normal distribution $\theta _i\sim {\mathcal {N}}(m_t, d_t)$. Denoting the indicator function for an event that action *i* was chosen at step *k* as *I*(*i*, *k*), we can write

36 $$\begin{aligned} (d^i_t)^{-1}&= (d^i_0)^{-1} + \sum _{k=1\wedge (t-w+1)}^{t} I(i, k) \delta ^{-1} \end{aligned}$$

37 $$\begin{aligned} m^i_t&= \frac{(d^i_0)^{-1}m^i_0 + \sum _{k=1\wedge (t-w+1)}^{t} I(i,k) \delta ^{-1} r_{k}}{(d^i_0)^{-1} + \sum _{k=1\wedge (t-w+1)}^{t}\delta ^{-1} I(i,k)}. \end{aligned}$$

If the denominator in (37) is 0 then $m^i_t=m^i_0$.

For the logistic model presented in Sect. 3.3, the posterior will again be approximated using a normal distribution $\theta \sim {\mathcal {N}}(m_t, d_t)$. Denoting the action at time step $k \in \{1,2,...,t\}$ as $a_k$

38 $$\begin{aligned} d_{t}&= \left( d_0^{-1} + S_t \right) ^{-1} \end{aligned}$$

39 $$\begin{aligned} m_{t}&= d_{t} \left( d_0^{-1} \mu _0 + S_t m + \sum _{k=1\wedge (t-w+1)}^{t} (y_{k} - \zeta ( m^T a_k)) a_k\right) , \end{aligned}$$

40 $$\begin{aligned} \text {with} \quad S_t :&= \sum _{k=1\wedge (t-w+1)}^{t} \zeta (m^T a_k)(1-\zeta ( m^T a_k)) a_ka_k^T. \end{aligned}$$

An alternative to the sliding-window method is the discounting approach (Kocsis and Szepesvári, 2006; Garivier and Moulines, 2008). Within the posterior update, the observations have weights which decay exponentially with time. The further in the past the observation is, the less impact it will have on the posterior. More approaches for tackling non-stationary include dynamic adjustments of the window size as well as mixtures of the approaches (Burtini et al., 2015).

### Decision Rules

We turn now to decision rules which will leverage the bandit models of the environment to compute the values of actions and consequently choose an action. We start with the Eps-Greedy algorithm (Sutton and Barto, 1998) - a basic decision rule often used for learning problems. Then, we describe a variation of Upper Confidence Bound (UCB) algorithm (Auer, 2002) which is one of the most popular bandit algorithms. For the following, we will assume that the action set is discrete and finite, with the number of actions equal to *K*. The *i*-th action will be denoted as $a^i$.

#### Eps-Greedy

The Eps-Greedy algorithm is parameterised by a single parameter $\varepsilon$. At each time step, with probability $\varepsilon$, the agent performs an exploration and chooses a random action with uniform probability. Otherwise, the agent chooses an action that maximises the expected reward.

At time step *t*, this rule outputs a probability distribution over the actions. Let $k_{\max }$ denote the number of actions which maximise the expected reward. Then:

41 $$\begin{aligned} {\mathbb {P}}(a_t=a^i) = \left\{ \begin{array}{ll} (1-\varepsilon )/k_{\max } + \varepsilon /K & \text {if }{\mathbb {E}}[r_t | a_t=x_i] = \max _j {\mathbb {E}}[r_t | a_t=x_j],\\ \varepsilon /K &\text {otherwise}. \end{array}\right. \end{aligned}$$

#### Upper Confidence Bound (UCB)

Upper Confidence Bound (UCB) algorithms, introduced by (Auer, 2002), employ the optimism principle, which asserts that the agent should act optimistically with respect to its uncertainty about the true values of actions. At each step, the algorithm selects the action with the highest upper confidence bound, which accounts for both the estimated reward and the uncertainty. In cases where multiple actions have the same highest upper confidence bound, the ties are broken randomly. By choosing actions with higher upper confidence bounds, the algorithm explores less certain options while also exploiting arms that are believed to have high rewards.

In this paper, we will use the UCB-Bayes variant from Kaufmann et al. (2012). This variant fits the Bayesian perspective to the multi-armed bandit problem that we employ here, and it will allow us to compare different bandit models. In Kaufmann et al. (2012), the authors propose that the value of an action $v^i_t$, i.e. the upper confidence bound, is calculated using quantiles of the reward distribution. We have

42 $$\begin{aligned} v_t^i := Q\left( 1- \frac{1}{t(\log T)^c}, \lambda ^i_{t-1} \right) , \end{aligned}$$

where $[0,1] \ni p \mapsto Q(p, \lambda ^i_t)$ denotes a quantile function of the posterior distribution $\lambda ^i_{t-1}$, *T* and *c* are fixed parameters. That is, the value of an action is such that

43 $$\begin{aligned} {\mathbb {P}}(r_t\le v_t^i | {\mathcal {F}}_{t-1}) = 1- \frac{1}{t(\log T)^c}. \end{aligned}$$

${\mathcal {F}}_{t-1}$ denotes the filtration associated with the learning process.

Since the action values are calculated using the distribution of the reward, the same calculation can be used for both algorithms that use the classical model and those that use the logistic model. Consequently, the comparison will be more meaningful and less dependent on hyperparameters. In our case, we will simplify the algorithm to choose a fixed quantile *q* instead of the expression $1- \frac{1}{t(\log T)^c}$. Then, the value of an action will be calculated using

44 $$\begin{aligned} v_t^i := Q\left( q, \lambda ^i_{t-1} \right) . \end{aligned}$$

Note that for the classical model, $$(d_t^i)^{-1}=0$$ implies that for $$q>0.5$$, the value $$v_t^i $$ is $$\infty $$. This consequently means that the action *i* has priority. The ties are broken randomly, which also includes the situation where two or more actions have “infinite” value.

### Joining the Model and the Decision Rule

Putting together the available bandit models with the decision rules, we compile a list of bandit algorithms.

1. Stationary, classic Eps-Greedy algorithm parametrised by $\varepsilon$,
2. Stationary, logistic Eps-Greedy algorithm parametrised by $\varepsilon$,
3. Stationary, classic UCB-Bayes algorithm parametrised by *q*,
4. Stationary, logistic UCB-Bayes algorithm parametrised by *q*,
5. Non-stationary, classic Eps-Greedy algorithm parametrised by $\varepsilon$ and window size *w*,
6. Non-stationary, logistic Eps-Greedy algorithm parametrised by $\varepsilon$ and window size *w*,
7. Non-stationary, classic UCB-Bayes algorithm parametrised by *q* and window size *w*,
8. Non-stationary, logistic UCB-Bayes algorithm parametrised by *q* and window size *w*.

The list above is limited and not reflective of the myriad of the available reinforcement learning algorithms. However, thanks to the simplicity of the bandit algorithms, it will allow us to analyse the numerical simulations in terms of

- The underlying bandit model,
- The decision rule.

Subsequently, the analysis will allow us to draw conclusions on how each of these aspects affects the performance of a bandit within the pricing environment.

## Pricing Environment Setup for the Numerical Experiments

Table 2 presents the parameters of the pricing environment used for the numerical experiments. In practice, one would use the available data to calibrate the parameters of the model used for simulation and review of the algorithm.

**Table 2:** Numerical parameters for the pricing environment

Parameter	Value
Time steps *T*	1000 / 2000
Number of agents *N*	2
Reservation margin $S^C$	1
Agent noise $\omega$	0.15
Agents’ correlation $\rho$	0.5
Noise $\tau$	0.13
Number of actions $N_a$	Varying
Available actions	$N_a$ equispaced points between 0.1 and 0.9

For the purpose of following numerical experiments, we are predominantly concerned that the environment can be accurately modelled by the logistic model used by the bandit algorithm. The logistic model error analysis in Sect. 3.3.1 has shown that this requirement is satisfied.

### Single-Stage Nash Equilibria

In Sect. 2.2, we proved that under Assumption 1 there exists a unique Nash equilibrium. This result is however a consequence of the continuous action space. When the action space is discretised, there might be two Nash equilibria located on the actions closest the unique Nash equilibrium point. As the number of actions increases, the computed Nash points will converge to the true, unique Nash equilibrium.

We computed the Nash equilibria for increasing number of actions for the environment parameters presented in Table 2. For all numbers of actions, there were two Nash equilibria, both of which were symmetric - both players quote the same margin and receive the same payoff per step. Table 3 presents the computed Nash equilibria. In the limit of increasing number of actions, the Nash equilibria appear to converge to a Nash strategy with margins 0.163 and payoffs 0.81.

**Table 3:** Nash equilibria vs number of actions for the discretization of the action space

Number of actions	First Nash equilibrium		Second Nash equilibrium	
	Margins	Payoffs	Margins	Payoffs
5	0.1	0.050	0.3	0.150
9	0.1	0.050	0.2	0.100
17	0.15	0.075	0.2	0.100
33	0.15	0.075	0.175	0.088
65	0.1625	0.081	0.175	0.088
129	0.1625	0.081	0.16875	0.085
801	0.162	0.081	0.163	0.081

In all cases, we have two Nash equlibria points which are neighbouring points on the action space grid

### Single-Stage Pareto Equilibria

A useful equilibrium definition, which will enhance the presentation of results, is a Pareto equilibrium. In this equilibrium, the agents collaborate to maximise the sum of their pay-offs. Proving uniqueness of the Pareto equilibrium is beyond the scope of this work. For the purpose of this paper, we will compute symmetric Pareto equilibrium, where each agent will commit to the same margin to maximise the sum of agent’s rewards. For the given numerical parameters and continuous action space, we have identified only a single symmetric Pareto equilibrium with agent payoff equal to approximately 0.364.

### Dimensionless Reward

Taking the Nash payoff as $r^N=0.081$ and the monopolistic payoff as $r^P=0.364$, we define a dimensionless one-step reward at time *t*, $\Delta _t$

45 $$\begin{aligned} \Delta _t := \frac{r_t-r^N}{r^P-r^N}. \end{aligned}$$

In the following, all rewards will be given in dimensionless quantities to enhance the interpretation of the results. $\Delta _t=0$ will indicate Nash payoffs, while $\Delta _t=1$ will correspond to monopolistic payoffs.

## Results

Section 5.1 presents tests of stationary algorithms against a fixed action agent, i.e. an agent who always quotes the same margin. As such, these tests provide a comparison between stationary classic and logistic models, as well as a means of choosing the hyperparameters for the bandit algorithms. Then, Sect. 5.2 presents tests of non-stationary algorithms against a non-stationary agent, i.e. agent which will periodically change their action. This will enable us to investigate the performance of the bandits in a non-stationary environment and, as before, an indication of the correct hyperparameters for non-stationary learning. Lastly, Sect. 5.3 presents simulations of games between algorithms. In these experiments, one agent is already within the environment and quotes the optimal margin. At time $t=0$, a new agent enters and starts learning, causing a sudden change in the environment and inducing “relearning” of the other agent. Therefore, these experiments show the resilience and adaptability of the algorithms in a realistic pricing scenario.

### Tests Against a Fixed-Action Agent

**Fig. 6:**
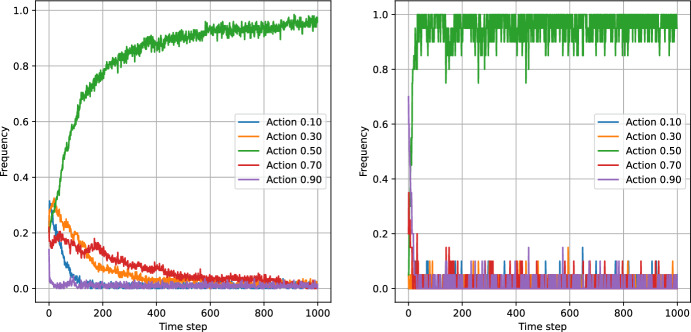
Eps-Greedy bandits action frequencies over the episode length. The frequency at given time step corresponds to the proportion of the simulations in which given action was taken. The left panel shows the action frequencies for the Eps-Greedy bandit with the classic model while the right panel shows the respective plot for the Eps-Greedy bandit with the logistic model

**Fig. 7:**
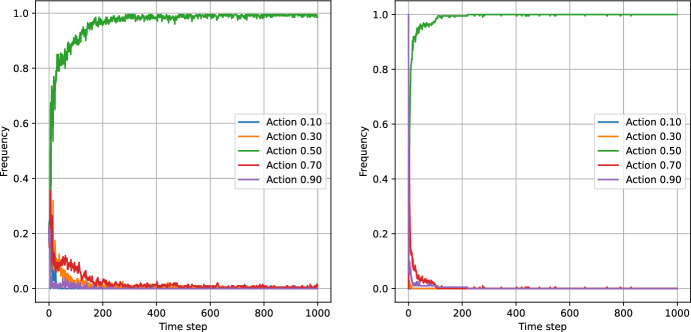
UCB-Bayes bandits action frequencies over the episode length. The frequency at given time step corresponds to the proportion of the simulations in which given action was taken. The left panel shows the action frequencies for the UCB-Bayes bandit with the classic model while the right panel shows the respective plot for the UCB-Bayes bandit with the logistic model

The test against a single agent involved 200 simulations of the game between a given bandit and a fixed-action agent. In this case, the fixed action agent margin was set at 0.7, and the number of actions was 5, for which the best response was 0.5. Figures 6 and 7 present the frequencies of actions versus time step obtained by averaging bandit actions over the 200 simulations. From the top, the figures show results for the Eps-Greedy algorithm and the UCB-Bayes algorithm. The left panels correspond to the classical agents and the right panels correspond to the logistic agents. The parameters for the algorithms were chosen to maximise the reward for the classical model. This resulted in $\varepsilon =0.05$ for the Eps-Greedy algorithm and $q=0.7$ for the UCB-Bayes algorithm. These parameters were fixed at these values for all subsequent numerical experiments.

In addition to the experiments above, we have simulated the same games with increased number of actions: $N_a \in \{5,9,17,33, 65, 129\}$. The action set consisted of $N_a$ equi-spaced points between 0.1 and 0.9. Table 4 presents the average cumulative rewards achieved by each type of bandit.

The table indicates that the logistic agents accumulate the highest reward, regardless of the number of actions or the algorithm. The reason behind the difference is the faster learning speed of the algorithms that use the logistic model. This is evident from the action frequency plots in Figs. 6 and 7 - the logistic bandits converge to the optimal action faster than their classical counterparts. From the inspection of Table 4, we can draw further conclusions. For the Eps-Greedy algorithm, increasing the number of actions increases the accumulated reward for both the classical and logistic models. The same is not true for the UCB algorithm. As the number of actions increases, so does the number of actions corresponding to low rewards. Because the classical model assumes reward independence, the algorithm must sample all these low-reward actions, which reduces the performance of the classical bandit. In contrast, since the logistic model captures the logistic relationship between the margin and the expected reward, the algorithm does not require sampling all actions to determine the optimal one. Therefore, as the number of actions increases, the learning speed - and consequently the performance of the agent - remain approximately unchanged.

We can concur that in a stationary environment, utilising prior information about the structure of the reward function can potentially provide competitive advantage to the pricing algorithm.

**Table 4:** Average cumulative rewards achieved by bandit algorithms in games against a fixed-action agent

Agent type	No. actions					
	5	9	17	33	65	129
Classic Eps-Greedy	1211	1208	1226	1221	1237	1242
Logistic Eps-Greedy	1261	1252	1283	1284	1288	1288
Classic UCB-Bayes	1292	1268	1249	1208	1158	1101
Logistic UCB-Bayes	1307	1288	1313	1315	1295	1315

Each cumulative reward corresponds to the bandit algorithm used (vertical axis) and number of available actions (horizontal axis)

### Tests of Non-Stationary Agents Against a Fluctuating Agent

We follow the previous experiments with tests of non-stationary algorithms against a fixed agent whose margin follows a periodic function. For $t \in \{1,2,3,4,...\}$:

46 $$\begin{aligned} s_t = \left\{ \begin{aligned}&0.3 \qquad \text { if } t-1 \text { mod } 500 <250\\&0.7 \qquad \text { otherwise. } \end{aligned} \right. \end{aligned}$$

Thus, the quoted margin forms a square wave with a low value 0.3, a high value 0.7, and a period 500. Thus, the agent induces a sudden change within the environment every 250 time steps. The number of actions was initially set to 5. The parameters $\varepsilon$ and *q* were fixed to 0.05 and 0.7 - the same as in the previous section. The number of time steps in the episode was still 1000. However, this time the performance metric was modified to include the accumulated reward from the last 500 steps. As such, the influence of the initial learning period was minimised, while retaining the history that encompasses a full period of opponent’s fluctuating action. The size of the sliding window was individually set for each of the algorithms to roughly maximise the accumulated reward from the last 500 steps. Table 5 presents the window sizes for each of the algorithms.

**Table 5:** Optimal sliding window size for bandit algorithms

Algorithm	Sliding window size
Non-stationary Classic Eps-Greedy $\varepsilon =0.05$	40
Non-stationary Logistic Eps-Greedy $\varepsilon =0.05$	40
Non-stationary Classic UCB-Bayes $q=0.7$	100
Non-stationary Logistic UCB-Bayes $q=0.7$	40

**Fig. 8:**
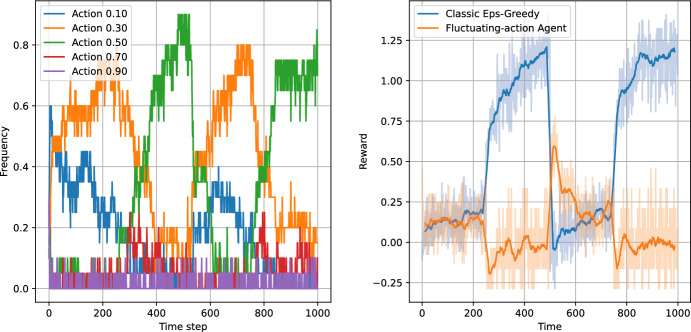
Test of non-stationary classic Eps-Greedy algorithm against a non-stationary agent. The left panel presents the action frequencies, i.e. the proportion of the simulations in which given action was taken at a time step. The right panel presents the rewards per step averaged across the simulations. The faded line shows the rewards, while the solid lines indicate the moving average with a 25 step window

**Fig. 9:**
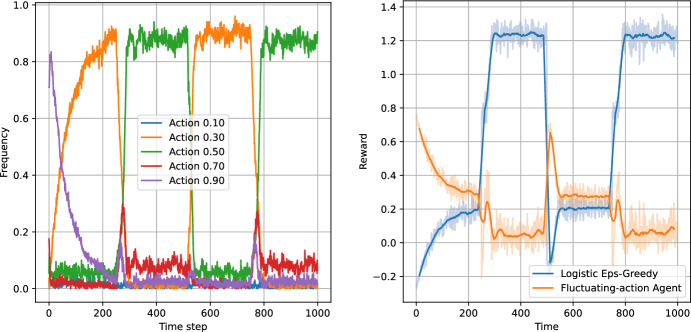
Test of non-stationary logistic Eps-Greedy algorithm against a non-stationary agent. The left panel presents the action frequencies, i.e. the proportion of the simulations in which given action was taken at a time step. The right panel presents the rewards per step averaged across the simulations. The faded line shows the rewards, while the solid lines indicate the moving average with a 25 step window

**Fig. 10:**
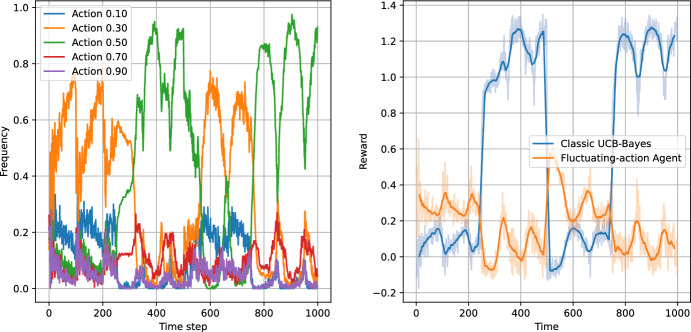
Test of non-stationary classic UCB-Bayes algorithm agains a non-stationary agent. The left panel presents the action frequencies, i.e. the proportion of the simulations in which given action was taken at a time step. The right panel presents the rewards per step averaged across the simulations. The faded line shows the rewards, while the solid lines indicate the moving average with a 25 step window

**Fig. 11:**
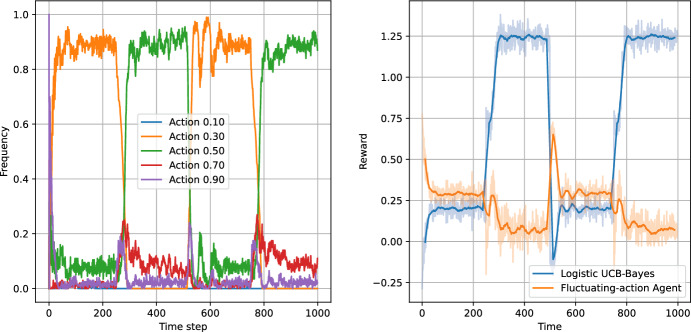
Test of non-stationary logistic UCB-Bayes algorithm against a non-stationary agent. The left panel presents the action frequencies, i.e. the proportion of the simulations in which given action was taken at a time step. The right panel presents the rewards per step averaged across the simulations. The faded line shows the rewards, while the solid lines indicate the moving average with a 25 step window

Figures 8, 9, 10 and 11 present the results of the algorithms tests with the number of actions $N_a=5$. The left panels present the frequencies of the actions, while the right panels present the moving average of average step rewards. The moving average was calculated using a 25 step window.

Table 6 presents the average cumulative rewards for the last 500 steps obtained in experiments with increased number of actions available to the algorithms: $N_a \in \{5,9,17,33, 65, 129\}$.

Both the action frequency and the reward history plots provide valuable information on the performance of the algorithms. Starting with the Eps-Greedy algorithm, we see that both classical and logistic bandits are able to adapt to the best action.

In the case of UCB algorithms, the classical variant, while achieving higher reward than the Eps-Greedy algorithm (first column of Table 6), shows signs of performance deterioration due to the optimism principle. After sampling a low-reward action, the algorithm retains this observation for only 100 steps, after which the uncertainty about the expected reward for the action significantly increases. Consequently, the optimism principle forces the UCB algorithm to sample the suboptimal action again. The same is not true for the logistic variant, which learns faster and does not need to re-sample all the actions.

The reward values in Table 6 indicate that increasing the number of actions slightly increases the performance of all algorithms except the classical UCB, where the increase in the action space deteriorates the performance of the algorithm.

The plots indicate that the better performance of the logistic bandits is caused again by the increased learning speed. Although the environment is changing, the changes are sudden and in-between the changes the environment stays stationary for 250 time steps, during which the logistic model approximates the environment well. Therefore, after each change, the bandits utilising the logistic model can adapt more quickly to the new reward function, and thus outperform their classical counterparts. We can conclude that in an environment which exhibits discrete and sparse variations, the use of prior information can provide an advantage to the learning algorithm.

**Table 6:** Average cumulative rewards from the last 500 steps of non-stationary algorithms in a game against a non-stationary agent

Agent type	No. actions					
	5	9	17	33	65	129
Classic Eps-Greedy $w=40$	281	290	297	298	297	297
Logistic Eps-Greedy $w=40$	334	333	338	339	338	339
Classic UCB-Bayes $w=100$	305	291	274	249	221	178
Logistic UCB-Bayes $w=40$	335	337	343	340	342	341

Each cumulative reward corresponds to the bandit algorithm used (vertical axis) and the number of available actions (horizontal axis)

### Tests with Concurrently Learning Agents

The last set of numerical experiments consists of simulations where one of the non-stationary agents is present in the environment (quotes the current optimal margin) and the other agent enters the environment and starts learning. Subsequent change in the environment induces the first agent to adjust its margin. Consequently, this creates a feedback loop with two concurrently learning algorithms. For this section, we decided to include only non-stationary Eps-Greedy algorithms with window size $w=40$. The UCB algorithms cannot be sensibly juxtaposed - the classical UCB deteriorates when the number of actions is increased and non-stationary UCB will always suffer from the optimism principle. For simplicity, the number of actions was set at 129. The higher the number of actions, the better the algorithms perform. Furthermore, the range of actions was extended to span [0, 0.9]. This was done to ensure that the optimal margin is well within the action space. When the optimal margin is close to the boundary of the action space, the potential advantage from the logistic model can diminish.

**Fig. 12:**
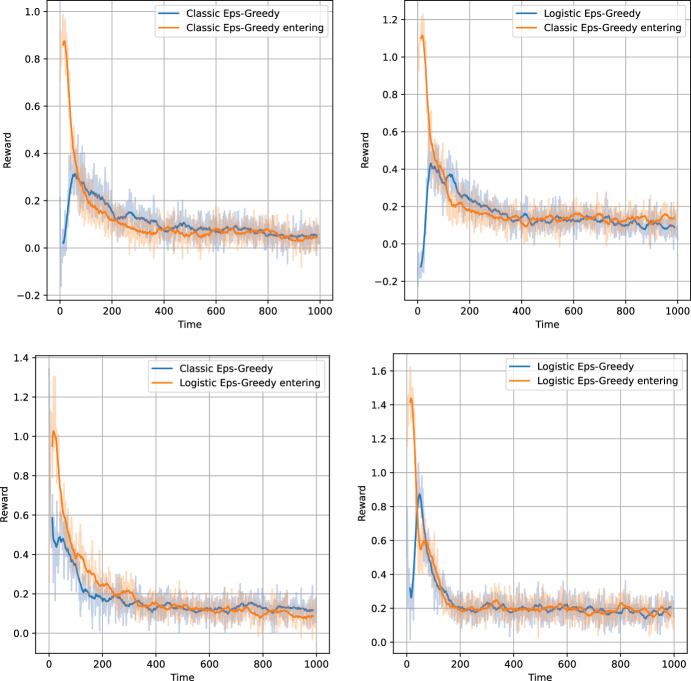
Average rewards over the episode for the market entering simulations with classic and logistic Eps-Greedy bandits. The faded lines indicate the reward (averaged across the simulations) while the solid lines indicate its moving average with a 25 step window. The top panels correspond to the simulations with the classic Eps-Greedy bandit entering the environment, while the bottom panels correspond to the simulations with the logistic Eps-Greedy bandit entering the environment. The panels on the left-hand side correspond to the simulations where the classic Eps-Greedy bandit is already present in the environment, while the panels on the right-hand side correspond to the simulations where the logistic bandit is already present in the environment

Figure 12 presents the average rewards throughout the simulation for different configurations of agents within and entering the environment. Table 7 presents the cumulative rewards achieved by the Eps-Greedy agents and the corresponding surplus obtained by the entering agent. Furthermore, the table displays the average reward per step averaged over the last 500 steps for a simulation with 2000 steps. This value can be interpreted as steady-state payoffs.

The first conclusion based on the left part of Table 7 is that the entering agent has an advantage over the agent that is already present within the environment. For all combinations of algorithms, the reward of the entering agent is the greatest. This is a result of a blank model which is able to learn quickly from the few initial observations, without being biased by past obsolete observations. This conclusion is further reinforced by the plots - the greatest per-step surplus is visible over the first steps. Afterward, the difference diminishes and can even be reversed.

In Sect. 5.2, we have shown that the logistic agents adapt faster. Based on Table 7 and Figs. 12, it is not possible to determine whether the increased learning speed has provided a competitive advantage to the logistic bandit. On the contrary, in terms of relative total reward, the performance of the classical Eps-Greedy bandit is slightly better.

The second column of Table 7 presents the steady-state pricing points to which the algorithms have converged. There is a noticeable difference between the setups. For games with classic algorithms only, the agents converge to prices close to the Nash prices - around 0.04 in terms of dimensionless reward. When a logistic bandit competes with a classical bandit, both algorithms quote higher margins and receive higher payoffs. When two logistic bandits compete, the steady-state pricing point is even higher, corresponding to around 0.2 in terms of dimensionless reward, which is considerably higher than the competitive pricing equilibrium.

After introduction of competition, the agents have lowered their margins towards the Nash equilibrium but clearly not exactly to the Nash equilibrium. What has caused this? For the classical algorithms, the main reason was the exploration rate. For the Eps-Greedy algorithms, $\varepsilon$ was around 0.05. Considering that the action space was mostly composed of margins higher than Nash, this means that the equilibrium margins have to be higher. This also contributed to the higher margins of the logistic algorithms, but does not fully explain the increased pricing inefficiency. We can speculate that the source of higher pricing originated in the non-stationarity of the logistic model. In the classical model, the reward for each arm is uncorrelated, and therefore forgetting past observations of action *i* does not influence the belief about action *i*. The same is not true for the logistic model. Forgetting past observations about margins higher than the optimal will infallibly cause the model to overestimate the offer acceptance probability, and thus the reward for these margins. Consequently, the non-stationarity of the logistic model will cause the algorithm to be overly optimistic.

This phenomenon could also be tied to the chosen prior. In this case, we used a prior with a large variance and a mean 0. This prior distribution spans logistic curves that are monotonically increasing, which is not suitable for the given monotonically decreasing probability curves.

**Table 7:** Results from games between Eps-Greedy bandits where one agent enters the environment with the other agent present

Case	Total reward			Final avg step reward	
	Entering	In	Difference	Entering	In
Classic enters Classic	121	108	+13	0.03	0.04
Classic enters Logistic	203	159	+44	0.13	0.13
Logistic enters Classic	206	176	+30	0.11	0.13
Logistic enters Logistic	267	237	+30	0.19	0.19

“Total reward” columns correspond to the total accumulated reward. Then, “entering” corresponds to the cumulative reward of the agent that enters the environment. “in” column corresponds to the reward of the agent already in the environment. “Final avg step reward” columns correspond to the average step rewards over the last 500 steps for the games with 2000 steps

### Further analysis of non-stationary equilibria

The previous result indicates that, for the given parameters, using non-stationary logistic model for approximating a non-stationary environment makes the bandit overly optimistic towards higher actions and creates an upward bias in the quoted margin. Consequently, this leads to an inefficient pricing equilibrium. In this section, we extend the analysis and check the robustness of this phenomenon. We run three additional sets of experiments explained in the following sections.

#### Increasing the Sliding Window $\tau$ of the Logistic Model

Firstly, we want to analyse how the pricing competition is influenced by the sliding window size $\tau$ of the logistic agent. Keeping all other parameters unchanged, we have conducted pricing competitions between the logistic agent and the classical agent and varied the $\tau$ of the logistic agent between 40 and 150. The sliding window of the classical agent was unchanged and equal to 40. These simulations included competitions where the classical agent was entering the environment with a converged logistic agent and vice versa, where the logistic agent was entering the environment with a converged classical agent.

Tables 8 and 9 present the results. Table 8 presents simulations where the logistic agent enters the environment and Table 9 corresponds to simulations where the classic agent enters the environment. The numbers represent average quantities averaged over the 100 simulation instances.

The results from Table 8 suggest that increasing the sliding window of the logistic agent does not significantly improve the performance of the logistic bandit when it enters the environment. Moreover, the results from Table 9 indicate that increasing the size of the sliding window seriously reduces the adaptability of the logistic agent - as the window size increases, the profit surplus of the classical agent from the first 1000 steps increases. At the same time, while increasing $\tau$ decreases the final payoffs and thus decreases the pricing inefficiency, it does not significantly improve the long-term performance of the logistic agent. The reward and margin values indicate that the logistic agent is still optimistic and, on average, quotes margins that are higher than optimal.

**Table 8:** Results from games between Eps-Greedy bandits where the logistic agent enters the environment with the classical agent already present

Logistic bandit	Total reward			Final avg step reward		Final avg step margin	
Sliding window $\tau$	Logistic (entering)	Classic (in)	Difference	Logistic (entering)	Classic (in)	Logistic (entering)	Classic (in)
40	183	181	+3	0.11	0.13	0.265	0.250
70	190	181	+9	0.10	0.11	0.250	0.241
100	191	174	+17	0.07	0.09	0.239	0.221
150	195	202	-7	0.07	0.08	0.234	0.227

Each row corresponds to a different value of the logistic agent’s sliding window size $\tau$. “Total reward” columns correspond to the total accumulated reward over 1000 steps. “Final avg step reward/margin” columns correspond to the average step rewards/margins over the last 500 steps for the games with 2000 steps. The margin at the Nash equilibrium was 0.163

**Table 9:** Results from games between Eps-Greedy bandits where the classical agent enters the environment with the logistic agent already present

Logistic bandit	Total reward			Final avg step reward		Final avg step margin	
Sliding window $\tau$	Classic (entering)	Logistic (in)	Difference	Classic (entering)	Logistic (in)	Classic (entering)	Logistic (in)
40	194	160	+34	0.13	0.13	0.260	0.274
70	220	162	+58	0.10	0.09	0.235	0.250
100	249	142	+107	0.10	0.07	0.227	0.243
150	321	149	+172	0.09	0.07	0.223	0.240

Each row corresponds to a different value of the logistic agent’s sliding window size $\tau$. “Total reward” columns correspond to the total accumulated reward over 1000 steps. “Final avg step reward/margin” columns correspond to the average step rewards/margins over the last 500 steps for the games with 2000 steps. The margin at the Nash equilibrium was 0.163

#### Adding Prior Information

**Fig. 13:**
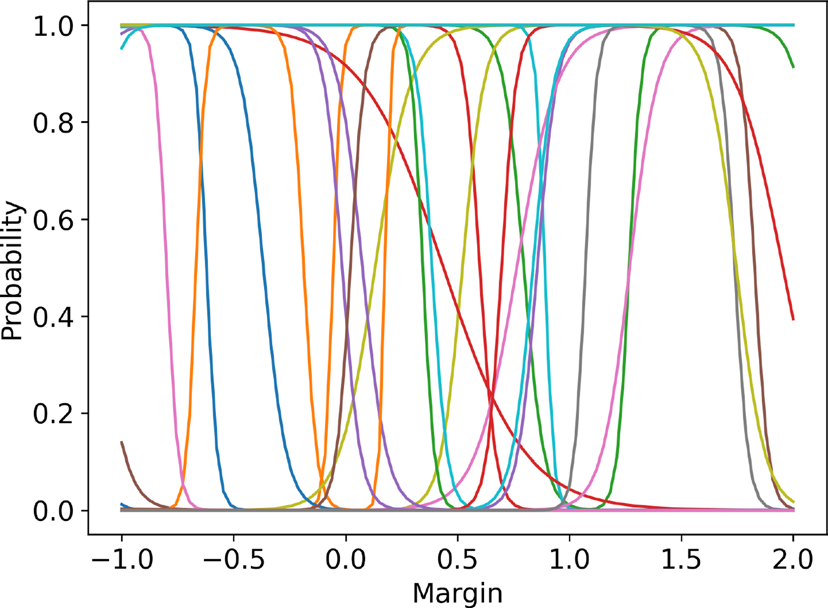
Logistic curves sampled from the initial prior

As previously mentioned, one of the possible sources of the upward bias is the prior on the model parameters. For the simulations performed, the prior was specified to be a normal distribution centred around 0 with a high variance. Figure 13 presents a number of logistic curves obtained by sampling the parameters from the prior. As can be seen in the figure, many of the logistic curves are increasing which is in clear violation of prior knowledge that the probability function is decreasing.

In the limit of high number of observations, the posterior probability that the logistic curve is increasing decreases towards 0 and does not influence the performance of the agent. In a non-stationary case, where the past observations are discarded, the posterior probability on the parameters corresponding to increasing probability functions remains significant. Consequently, the model overestimates the acceptance probability, which naturally increases the rewards for higher margins, leading to an upward bias in the logistic agent’s action.

In this section, we implement modifications to the prior so that it favours parameters that correspond to monotonically decreasing logistic curves. Using the new prior, we run the simulations of the pricing competition between the logistic agent and the classic agent to determine whether the modification successfully decreases the bias of the logistic agent. With the exception of the prior, all other parameters remain unchanged.

The prior for the logistic model was modified by feeding artificial observations to the model. Specifically, the model is given a fixed set of observations that convey the information that the function is decreasing. At each update of the model, the new posterior is calculated using the union of the artificial observations and the observations gathered from experience. While the number of observations from experience is capped at $\tau$ (sliding window size), the artificial observations are not discarded.

**Fig. 14:**
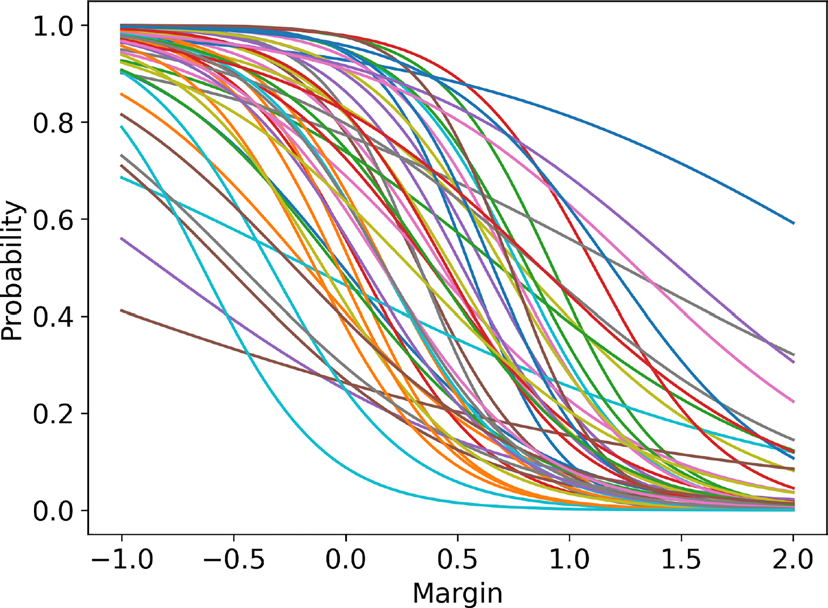
Logistic curves corresponding to samples from prior with artificial observations at left- and right-hand side of the action set

**Fig. 15:**
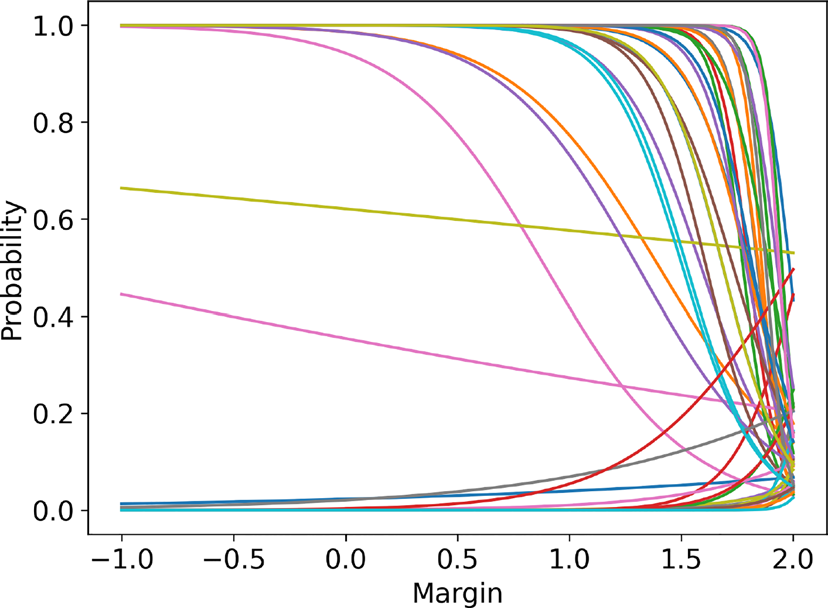
Logistic curves corresponding to samples from prior with artificial observations at right-hand side of the action set

For the simulations, we used two variants of the artificial observations. In the first variant, previous observations included a total of 10 observations, 5 corresponding to the margin $S=-1$ and 5 corresponding to margin $S=2$. The observations at $S=-1$ specified that the offer was accepted, and the observations at $S=2$ specified that the offer was not accepted. This variant is referred to as “both”. In the second variant, the artificial observations only included 5 observations at the margin $S=2$ - the variant is referred to as “right”. Figures 14 and 15, respectively, present the logistic curves sampled from the modified priors. While there are still functions that are increasing, the monotonically decreasing functions dominate, as was desired. Although the priors might be in conflict with some values of the opponent margin, they do agree with the probability curve at the Nash equilibrium.

Given the new priors, we repeated the tests with the concurrently learning agents. Table 10 presents the results of the simulations in which the logistic agent enters the environment with a fully converged classic agent and starts learning. Table 11 shows the results of the simulations in which the classic agent enters the environment with a fully converged logistic agent and starts learning.

**Table 10:** Results from games between Eps-Greedy bandits where the logistic agent enters the environment with the classical agent already present

Model prior	Total reward			Final avg step reward	
Variant	Logistic (entering)	Classic (in)	Difference	Logistic (entering)	Classic (in)
“both”	213	255	$-$42	0.11	0.21
“right”	221	273	$-$52	0.15	0.23

Each row corresponds to the variant of the prior used by the logistic agent. “Total reward” columns correspond to the total accumulated reward over 1000 steps. “Final avg step reward” columns correspond to the average step rewards over the last 500 steps for the games with 2000 steps

**Table 11:** Results from games between Eps-Greedy bandits where the classical agent enters the environment with the logistic agent already present

Model prior	Total reward			Final avg step reward	
Variant	Classic (entering)	Logistic (in)	Difference	Classic (entering)	Logistic (in)
“both”	266	174	+92	0.22	0.12
“right”	274	175	+97	0.24	0.14

Each row corresponds to the variant of the prior used by the logistic agent. “Total reward” columns correspond to the total accumulated reward over 1000 steps. “Final avg step reward” columns correspond to the average step rewards over the last 500 steps for the games with 2000 steps

The results of the numerical experiments indicate that modifying the prior only worsened the performance of the logistic agent. The logistic agent no longer has the advantage of being the agent entering the environment. Furthermore, the long-term average payoffs indicate that the pricing inefficiency is worse and that the upward bias of the logistic agent increased relative to the base simulations. The upward bias of the logistic model cannot be easily mitigated by modifications of the prior.

#### Varying the Environment

**Fig. 16:**
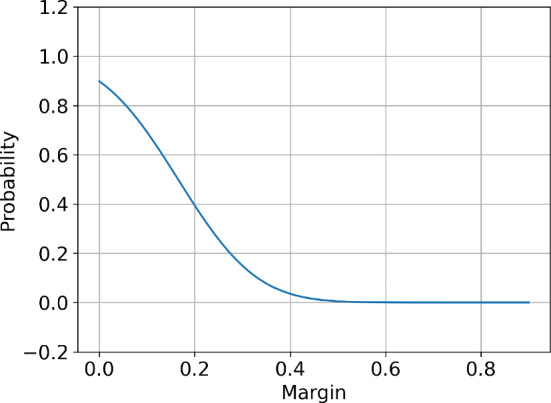
Probability function at the Nash equilibrium for $\omega =0.15$

**Fig. 17:**
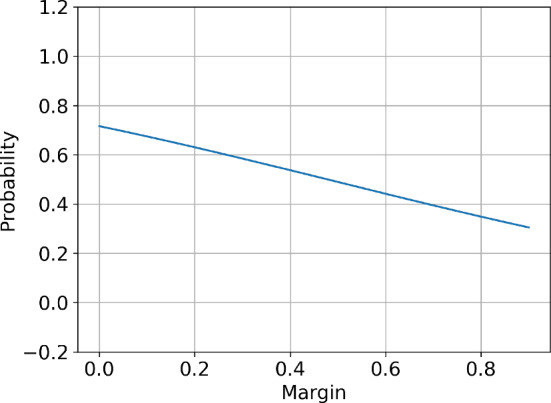
Probability function at the Nash equilibrium for $\omega =1.0$

In the last set of experiments, we check whether the observed pricing equilibrium is a result of the specific environment parameters. The environment is set up using 4 parameters: noise $\omega ^2$, correlation $\rho$, reservation noise $\tau ^2$ and mean reservation margin $S^C$. Of these parameters, the one that influences the probability function and the competition between agents is the noise parameter $\omega ^2$. Figures 16 and 17 show the probability functions for $\omega =0.15$ and $\omega =1.00$ respectively. The smaller the $\omega$ the sharper the drop in the probability as the margin increases. Due to the importance of the parameter, the last set of experiments analyses the effect of the noise $\omega ^2$ on the pricing equilibrium to which the logistic and classic agents converge. Table 12 presents the results of the simulations in which the logistic agent enters the environment with a fully converged classic agent and starts learning. Table 13 shows the results of the simulations in which the classic agent enters the environment with a fully converged logistic agent and starts learning.

From the results, we can draw two conclusions. Firstly, for every tested value of $\omega$, the logistic agent’s margin is higher than the classical agent’s margin which means that the upward bias observed in the baseline experiments is still present if we change the environment. The second conclusion regards the pricing level at the non-stationary equilibrium. We observe that as $\omega$ increases, the long-term per-step reward decreases. When $\omega$ is higher, the margin at the Nash equilibrium is higher - this is evident from inspection of the second column of Table 12. Since the action set of the agents is fixed to the range [0., 0.9], when the margin at the equilibrium increases, the actions explored by the epsilon greedy agents increasingly correspond to the margins below the Nash equilibrium. Thus, the exploration effectively decreases the mean margin quoted by the agents. This is then further amplified by the feedback effect, as a lower average margin results in lower optimal response.

**Table 12:** Results from games between Eps-Greedy bandits where the logistic agent enters the environment with the classical agent already present

Agent	Nash	Average margin		Final avg step reward	
Noise $\omega$	Margin	Logistic (entering)	Classic (in)	Logistic (entering)	Classic (in)
0.10	0.108	0.239	0.210	0.11	0.15
0.15	0.163	0.265	0.250	0.11	0.13
0.30	0.319	0.400	0.395	0.11	0.12
0.50	0.418	0.551	0.524	−0.6	0.07
0.70	0.606	0.645	0.574	−0.21	0.00
1.00	0.783	0.737	0.637	−0.49	−0.39

Each row corresponds to a different value of agent noise $\omega$. The column “Nash margin” shows the Nash margin for the given environment parameter. The last two columns present the normalised long-term step rewards. Columns “Average margin” display the long-term margins quoted by the agents. The margins and per-step rewards are the average values averaged over last 500 steps of a 2000 step long episode and over 100 simulation instances

**Table 13:** Results from games between Eps-Greedy bandits where the classical agent enters the environment with the logistic agent already present

Agent	Nash	Average margin		Final avg step reward	
Noise $\omega$	Margin	Classic (entering)	Logistic (in)	Classic (entering)	Logistic (in)
0.10	0.108	0.208	0.237	0.15	0.11
0.15	0.163	0.260	0.273	0.13	0.13
0.30	0.319	0.400	0.402	0.13	0.13
0.50	0.418	0.523	0.554	0.10	−0.06
0.70	0.606	0.592	0.663	0.05	−0.14
1.00	0.783	0.636	0.722	−0.50	−0.45

Each row corresponds to a different value of agent noise $\omega$. The column “Nash margin” shows the Nash margin for the given environment parameter. The last two columns present the normalised long-term step rewards. Columns “Average margin” display the long-term margins quoted by the agents. The margins and per-step rewards are the average values averaged over last 500 steps of a 2000 step long episode and over 100 simulation instances

## Conclusions

In the first part of this work, we introduced a pricing environment. In the derivation of the environment, we started from an analysis of a single auction and computed the probability and expected reward functions given the pricing margins of all market participants. Based on the analysis of those functions, we have put forward assumptions about the expected reward functions. Given these, we proved the existence and uniqueness of a Nash equilibrium for a single-stage pricing game induced by the environment. Furthermore, in an infinite-horizon game where all agents play best response to the previous stage margins, we proved that the strategies will converge to the unique Nash equilibrium.

In the second part of the paper, we presented numerical experiments of bandit algorithms within the introduced pricing environment. The bandit algorithms were differentiated in terms of the decision rule, the used model of the environment, and the non-stationarity assumption. In the process, we have also developed a logistic model that efficiently approximates the reward function of the pricing environment.

The experiments have shown that the application of the logistic model effectively increases the speed with which bandits learn when the environment is fixed. Thus, in a stationary environment, where the opponent keeps their strategy fixed, this increased speed of learning will likely increase the total reward accumulated by the agent. In a non-stationary environment, which experiences sudden changes but stays stationary in between, the increased efficiency of learning will again increase the total reward.

When the environment is fixed, it can be accurately approximated by the logistic model, and then the addition of the model to the bandit algorithm can potentially enhance the performance of the agent.

The same is not true for situations where two bandit algorithms learn simultaneously within the same environment. Numerical experiments of competitions between bandit algorithms have shown that bandits that employed the logistic model had no advantage over bandits that used the classical model of the environment. Furthermore, the combination of the non-stationarity and the logistic model led to unfounded optimism and consequently inefficient pricing. This phenomenon is robust and cannot be easily mitigated by increasing the sliding window size or through modifications of the prior.

When two or more agents learn, the environment, as perceived by a single agent, is constantly changing, and thus cannot be faithfully represented by the logistic model. Consequently, the logistic model is no longer likely to improve the performance of the bandit. In order to improve the competitiveness of learning algorithms using prior information, one has to include an accurate representation of the opponents within the model of the environment. If that is not possible due to complexity or lack of information, opting for a simple algorithm is a good option.

### Further Work

An interesting path for future work would include analysis of the relationship between the model of the environment and the speed with which the agent learns its parameters. This could point to the specific elements of environment modelling that benefit the performance of learning agents. Another direction of research could involve designing a dynamic environment that could include the fact that opposing agents continuously change over time. Lastly, the study could be extended to include state-aware learning algorithms such as Q-learning (Watkins and Dayan, 1992) or Soft Actor-Critic (Haarnoja et al., 2018).

## Data Availability

Numerical experiments presented in this work have been implemented in Python. All the code necessary to reproduce the results can be accessed at the Github repository https://github.com/ls2716/Competetive_Pricing_Using_Model-Based_Bandits.

## Appendix A: Proofs of lemmas

### A.1. Proof of Lemma 1

#### Proof

We can write for the expected reward over a single step

47 $$\begin{aligned} {\mathbb {E}}\left[ R^i | S, S^C\right] = {\mathbb {E}}\left[ S^i \mathbbm {1}_{S^i + V^i< \min _{j\ne i} (S^j + V^j)} \mathbbm {1}_{S^i + V^i < P^C} | S,S^C \right] \end{aligned}$$

Let $\xi ^{0},...,\xi ^{N},\xi ^C$ be independent standard normal random variables such that

48 $$\begin{aligned} V^i-M= &  \omega \sqrt{\rho } \xi ^{0} + \omega \sqrt{1-\rho }\xi ^{i}, \end{aligned}$$

49 $$\begin{aligned} P^C-M= &  S^C + \tau \xi ^C. \end{aligned}$$

Let us further rewrite the expected reward

50 $$\begin{aligned} \begin{aligned} {\mathbb {E}}\left[ R^{i}\Big | S, S^C\right]&= {\mathbb {E}}\left[ S^i\mathbbm {1}_{S^i + V^i< \min _{j\ne i} (S^j + V^j)} \mathbbm {1}_{S^i + V^i< P^C} \Big | S, S^C \right] \\&= {\mathbb {E}}\left[ S^i \mathbbm {1}_{S^i + V^i -M< \min _{j\ne i} S^j + V^j-M} \mathbbm {1}_{S^i + V^i -M < P^C -M} \Big | S, S^C \right] . \end{aligned} \end{aligned}$$

Now, let us focus on the events in the indicator functions. Using random variables $\xi$, we have

51 $$\begin{aligned} \begin{aligned}&\Big ( S^i + V^i -M< \min _{j\ne i} (S^j + V^j -M ) \Big ) = \\&\quad =\left( S^i + \omega \sqrt{\rho } \xi ^{0} + \omega \sqrt{1-\rho }\xi ^{i}< \min _{j\ne i} (S^j + \omega \sqrt{\rho } \xi ^{0} + \omega \sqrt{1-\rho }\xi ^{j})\right) \\&\quad =\left( S^i + \omega \sqrt{1-\rho }\xi ^{i}< \min _{j\ne i} (S^j + \omega \sqrt{1-\rho }\xi ^{j})\right) \\&\quad = \bigcap _{i\ne i} \left( S^i + \omega \sqrt{1-\rho }\xi ^{i} < (S^j + \omega \sqrt{1-\rho }\xi ^{j})\right) . \end{aligned} \end{aligned}$$

Fixing $\xi ^i$ as *x*, we can write for a given *j*

52 $$\begin{aligned} \begin{aligned}&{\mathbb {P}}\Big (S^i + \omega \sqrt{1-\rho }\xi ^i< (S^j + \omega \sqrt{1-\rho }\xi ^{j})\ \Big | \ \xi ^i=x\Big ) = \\&\quad = {\mathbb {P}}\left( - \omega \sqrt{1-\rho }\xi ^{j} < -(S^i - S^j + \omega \sqrt{1-\rho }\xi ^i)\ \Big |\ \xi ^i=x\right) \\&\quad = \Phi \left( -\frac{S^{i} - S^{j} +\omega \sqrt{1-\rho }x}{\omega \sqrt{1-\rho }}\right) , \end{aligned} \end{aligned}$$

where $\Phi$ is a normal cdf with $\varphi$ as the associated pdf. We assume that none of the denominators is 0, that is, $\tau >0$, $\omega >0$, and $\rho <1$. Similarly, for the other indicator function, we have

53 $$\begin{aligned} \begin{aligned}&\Big ( S^i + V^i -M< P^C -M \Big ) = \\&\quad =\left( S^i + \omega \sqrt{\rho } \xi ^{0} + \omega \sqrt{1-\rho }\xi ^{i} < S^C+\tau \xi ^C \right) . \end{aligned} \end{aligned}$$

Fixing $\xi ^i$, we can write

54 $$\begin{aligned} \begin{aligned}&{\mathbb {P}}\Big (S^i + \omega \sqrt{\rho } \xi ^{0} + \omega \sqrt{1-\rho }\xi ^{i}< S^C+\tau \xi ^C\ \Big | \ \xi ^i = x \Big ) = \\&\quad = {\mathbb {P}}\left( - \tau \xi ^C + \omega \sqrt{\rho } \xi ^{0} < -(S^i - S^C + \omega \sqrt{1-\rho }\xi ^i)\ \Big | \ \xi ^i = x \right) \\&\quad = \Phi \left( -\frac{S^{i} - S^{C} +\omega \sqrt{1-\rho }x}{\sqrt{\tau ^2 + \rho \omega ^2}}\right) . \end{aligned} \end{aligned}$$

With $\xi ^i$ and $\xi ^0$ fixed, indicator variables

55 $$\begin{aligned} &  \mathbbm {1}{\Big (S^i + \omega \sqrt{1-\rho }\xi ^i < (S^j + \omega \sqrt{1-\rho }\xi ^{j})\Big )}, \end{aligned}$$

56 $$\begin{aligned} &  \mathbbm {1}{\Big (S^i + \omega \sqrt{\rho } \xi ^0 + \omega \sqrt{1-\rho }\xi ^i < S^C + \tau \xi ^C\Big )} \end{aligned}$$

and $S^i$ are independent and thus we can write for the expected reward

57 $$\begin{aligned} \begin{aligned}&{\mathbb {E}}\left[ R^{i}\Big | S, S^C\right] = {\mathbb {E}}\left[ S^i \mathbbm {1}_{S^i + V^i< \min _{j\ne i} (S^j + V^j)} \mathbbm {1}_{S^i + V^i < P^C} \Big | S, S^C \right] \\&\quad = S^i \int _{\mathbb {R}} \Phi \left( -\frac{S^{i} - S^{C} +\omega \sqrt{1-\rho }x }{\sqrt{\tau ^2 + \rho \omega ^2}}\right) \prod _{j\ne i} \Phi \left( -\frac{S^i - S^{j} +\omega \sqrt{1-\rho }x}{\omega \sqrt{1-\rho }}\right) \varphi (x)dx. \end{aligned} \end{aligned}$$

Similarly, we can calculate the probability of the offer acceptance (the proposed price is the minimum and lower than the reservation price), as

58 $$\begin{aligned} \begin{aligned}&{\mathbb {E}}\left[ \mathbbm {1}_{S^i + V^i< \min _{j\ne i} (S^j + V^j)} \mathbbm {1}_{S^i + V^i < P^C} \Big | S, S^C \right] \\&\quad = \int _{\mathbb {R}} \Phi \left( -\frac{S^{i} - S^{C} +\omega \sqrt{1-\rho }x }{\sqrt{\tau ^2 + \rho \omega ^2}}\right) \prod _{j\ne i} \Phi \left( -\frac{S^{i} - S^{j} +\omega \sqrt{1-\rho }x}{\omega \sqrt{1-\rho }}\right) \varphi (x) dx. \end{aligned} \end{aligned}$$

$$\square $$

### A.2. Proof of Lemma 2

#### Proof

For what follows, the vector and matrix entries will be indexed using a superscript, using a coma if a variable already has a superscript. For example, $B(S_b)^i$ denotes the *i*-th entry of the vector $B(S_b)$. Without loss of generality, let us take any *i* and let us have $B(S_a)^i > B(S_b)^i$.

Now, let’s define

59 $$\begin{aligned} D^i&:= \max _{j \ne i} \Big (S_a^{j} - S_b^{j} \Big ). \end{aligned}$$

$D^i$ is the maximum difference between the corresponding entries in the vectors $S_a$ and $S_b$. From monotonicity in Assumption 1, since $B(S_a)^{i} > B(S_b)^{i}$ we have $D^i>0$. Furthermore, let’s define a new set of margins $S_b'$, such that

60 $$\begin{aligned} S_b'^{j} = \left\{ \begin{aligned} S_b^{j} \quad&\text {if } j=i \\ S_a^{j} \quad&\text {if } S_a^{j}\ge S_b^{j} \\ S_b^{j} \quad&\text {if } S_a^{j}< S_b^{j} \end{aligned}\right. \end{aligned}$$

That is, when constructing $S_b'^{j}$, we only increase the elements of $S^{j}_b$ where $i\ne j$ and the corresponding entry was greater in $S_a$ than in $S_b$. Then, from sublinearity in Assumption 1

61 $$\begin{aligned} B(S_b')^{i} \le B(S_b)^{i} + \beta D^i, \end{aligned}$$

where $\beta \in (0,1)$. Furthermore, from monotonicity in Assumption 1

62 $$\begin{aligned} B(S_b')^{i} > B(S_a)^{i}. \end{aligned}$$

This is because we have that for all $i\ne j$, $S_b'^j \ge S_a^j$. Thus,

63 $$\begin{aligned} B(S_b)^{i} + \beta D^i&\ge B(S_a)^{i} \end{aligned}$$

64 $$\begin{aligned} \beta D^i&\ge B(S_a)^{i} - B(S_b)^{i} \end{aligned}$$

Since we assumed that $B(S_a)^{i} > B(S_b)^{i}$, we have

65 $$\begin{aligned} \beta D^i \ge \big |B(S_a)^{i} - B(S_b)^{i}\big | \end{aligned}$$

Consequently

66 $$\begin{aligned} \big |B(S_a)^{i} - B(S_b)^{i}\big | \le \beta \max _{j \ne i} \Big (S_a^{j} - S_b^{j}\Big ) \le \beta \max _{j} \big |S_a^{j} - S_b^{j}\big | = \beta \big |S_a - S_b \big |_\infty . \end{aligned}$$

The index *i* was chosen without the loss of generality, and thus this concludes the proof. $\square$

## Appendix B: Proofs of Theorems 3 and 4

### Proof

For any strategy $\pi ^i$, let us define

67 $$\begin{aligned} S_u(\pi ^i)&= \inf \{ s \in {\mathbb {R}} \cup \{\infty \} : \pi ^i([s,\infty )])=0\} \end{aligned}$$

68 $$\begin{aligned} S_l(\pi ^i)&= \sup \{ s \in {\mathbb {R}} \cup \{-\infty \} : \pi ^i([-\infty ,s)])=0\} \end{aligned}$$

Thus, for a given strategy $\pi ^i$, $S_u(\pi ^i)$ is the smallest number such that the probability of quoting margin from $[S_u(\pi ^i), \infty ]$ is 0 and $S^i_l$ is the highest number such that the probability of quoting margin from $[-\infty , S^i_l]$ is 0. Furthermore, let us define vectors $S_u(\pi ) = [S_u(\pi ^1), S_u(\pi ^2),...,S_u(\pi ^3)]$ and $S_l(\pi ) = [S_l(\pi ^1), S_l(\pi ^2),...,S_l(\pi ^N)]$.

By inspection, since the reward for negative margins is negative and for all *i*

69 $$\begin{aligned} \lim _{S^i\rightarrow \infty } \lim _{S^{(-i)}\rightarrow \infty } {\mathbb {E}}\left[ R^i | S^i, S^{(-i)}\right] = 0. \end{aligned}$$

Then, for any $\pi$

70 $$\begin{aligned} \max _i B(S_u(\pi ))&< \infty , \end{aligned}$$

71 $$\begin{aligned} \min _i B(S_l(\pi ))&> -\infty . \end{aligned}$$

Thus, for simplicity, we will assume from now on that all $S^i_u$ and $S^i_l$ will be finite.

Now, let us take a vector of mixed strategies $\pi ^i$ and one of the corresponding best response strategy vectors $\pi '$ where for all *i*, ${\pi '}^{i}$ is a best response to $\pi ^{(-i)}$.

Under Assumption 1, for any agent *i*, the expected reward function has a unique maximiser, which is an increasing function of the opponents’ margins. Thus, for all *i*

72 $$\begin{aligned} S_u(\pi ')^i \le B(S_u(\pi ))^i \end{aligned}$$

Similarly, by the same arguments, we get that for all *i*

73 $$\begin{aligned} S_l(\pi ')^i \ge B(S_l(\pi ))^i. \end{aligned}$$

For the *i*-th player, the support of the new policy is upper and lower bounded by the best response function applied to the bounds of the supports of opponent policies.

Thus, by induction, after *n* consecutive best response plays, we can write for the resulting vector of agents strategies $\pi _n$ and for all *i*

74 $$\begin{aligned} S_u(\pi _n)^i \le \left[ (B)^n (S_u(\pi ))\right] ^i \end{aligned}$$

and

75 $$\begin{aligned} S_l(\pi _n)^i \ge \left[ (B)^n (S_l(\pi ))\right] ^i, \end{aligned}$$

where $(B)^n$ denotes *n* applications of the transform *B*. The *i*-th agent policy support will be upper and lower bounded by repeated application of best response transform to the upper and lower bounds of the starting policy.

By Lemma 2, *B* is a contraction with a fixed point $S^*$ - the vector of margins that corresponds to the unique pure Nash equilibrium. Thus

76 $$\begin{aligned} \lim _{n\rightarrow \infty } S_l(\pi _n) = \lim _{n\rightarrow \infty } S_u(\pi _n) = S^* \end{aligned}$$

and $\pi ^n$ converges in distribution to the unique pure Nash equilibrium. $\square$

## Appendix C: Alternative Pricing Environment

In the previous section, the market environment was derived bottom-up from the value estimates of the items and the quoted margins. Consequently, this resulted in a reward function that includes a mixture of normal cdfs and which is difficult to analyse.

An alternative option for the environment would be to use a slight modification of the pricing environment used in Calvano et al. (2020). In this setup, there are *N* agents / players with quality indices $\{a_i\}_{i=1}^N$ and costs $\{c_i\}_{i=1}^N$. At each time step, the agents propose prices $\{p_i\}_{i=1}^N$. In our notation, the agents set margins $\{s_i\}_{i=1}^N$ instead of prices - one can be calculated from the other using the relation $s^i=p_i-c_i$. Then, given the margins, the probabilities of offer acceptance $\{q_i\}_{i=1}^N$ are calculated as

77 $$\begin{aligned} q_i(s^1, s^2, ..., s^N) = \frac{\exp \left( \frac{a_i-c_i-s^i}{\mu } \right) }{\sum _{j=1}^N \exp \left( \frac{a_j-c_j-s^j}{\mu }\right) + \exp \left( \frac{a_0}{\mu } \right) }, \end{aligned}$$

where $\mu$ and $a_0$ are the environment parameters. If *i*-th agent’s offer is accepted, the agent receives a reward $s^i$. Thus, the expected reward can be written as

78 $$\begin{aligned} r_i(s^1, s^2, ..., s^N) = q_i(s^1, s^2, ..., s^N) \cdot s^i. \end{aligned}$$

Note that in Calvano et al. (2020), the probability of offer acceptance is regarded as demand at the price level. However, this quantity is bounded between 0 and 1 and can therefore be interpreted as probability. Moreover, the expected reward is unchanged between these two interpretations.

### Theorem 5

The expected reward function (78) satisfies Assumption 1.

This indicates that in the environment setup above there exists a unique Nash equilibrium and that the best response transform is contractive.

### C.1. Proof of Theorem 5

#### Proof

For conciseness, we define:

79 $$\begin{aligned} b_i = a_i- c_i. \end{aligned}$$

Then, for *i*-th agent, the offer acceptance probability is

80 $$\begin{aligned} q_{i} = \frac{\exp \left( \frac{b_i - s^{i}}{\mu } \right) }{\sum _{j=1}^N\exp \left( \frac{b_{j} - s^{j}}{\mu } \right) + \exp \left( \frac{a_0}{\mu } \right) }, \end{aligned}$$

and the expected reward is $r_i = q_i s^i$.

Expanding the probability $q_i$, we get

81 $$\begin{aligned} r_{i} = \frac{\exp \left( \frac{b_i - s^{i}}{\mu } \right) s^i}{\sum _{j=1}^N\exp \left( \frac{b_{j} - s^{j}}{\mu } \right) + \exp \left( \frac{a_0}{\mu } \right) }. \end{aligned}$$

We take the derivative of the reward with respect to the margin $s^i$:

82 $$\begin{aligned} \frac{\partial r_i}{\partial s^i}&= \frac{\exp \Big (\frac{b_{i} - s^i}{\mu }\Big )\left( 1 - s^i/\mu \right) \left[ \sum _{j=1}^N\exp \Big (\frac{b_{j} - s^{j}}{\mu } \Big ) + \exp \Big (\frac{a_0}{\mu } \Big )\right] }{\left[ \sum _{j=1}^N\exp \Big (\frac{b_{j} - s^{j}}{\mu } \Big ) + \exp \Big (\frac{a_0}{\mu } \Big )\right] ^2} \end{aligned}$$

83 $$\begin{aligned}&\quad + \frac{ \exp \Big (\frac{b_{i} - s^i}{\mu }\Big ) \exp \Big (\frac{b_{i} - s^i}{\mu }\Big ) s^i /\mu }{\left[ \sum _{j=1}^N\exp \Big (\frac{b_{j} - s^{j}}{\mu } \Big ) + \exp \Big (\frac{a_0}{\mu } \Big )\right] ^2}. \end{aligned}$$

Simplifying, we get

84 $$\begin{aligned} \frac{\partial r_i}{\partial s^i} = \frac{\exp \Big (\frac{b_{i} - s^i}{\mu }\Big )\left( B_i + \exp \Big (\frac{b_{i} - s^i}{\mu }\Big ) - s^i B_i/\mu \right) }{A^2}, \end{aligned}$$

where

85 $$\begin{aligned} A := \sum _{j=1}^N\exp \Big (\frac{b_{j} - s^{j}}{\mu } \Big ) + \exp \Big (\frac{a_0}{\mu } \Big ) \end{aligned}$$

and

86 $$\begin{aligned} B_i := A - \exp \Big (\frac{b_{i} - s^i}{\mu }\Big ) = \sum _{j\ne i}^N\exp \Big (\frac{b_{j} - s^{j}}{\mu } \Big ) + \exp \Big (\frac{a_0}{\mu } \Big ). \end{aligned}$$

By inspection, $B_i$ is constant w.r.t. $s^i$.

The function will achieve its maximum when

87 $$\begin{aligned} B_i + \exp \Big (\frac{b_{i} - s^i}{\mu }\Big ) - s^i B_i/\mu = 0. \end{aligned}$$

By inspection

88 $$\begin{aligned} \lim _{s^i\rightarrow -\infty } \left[ B_i + \exp \Big (\frac{b_{i} - s^i}{\mu }\Big ) - s^i B_i/\mu \right]= &  +\infty , \end{aligned}$$

89 $$\begin{aligned} \lim _{s^i\rightarrow +\infty } \left[ B_i + \exp \Big (\frac{b_{i} - s^i}{\mu }\Big ) - s^i B_i/\mu \right]= &  -\infty \end{aligned}$$

and

90 $$\begin{aligned} \frac{\partial }{\partial s^i} \left[ B_i + \exp \Big (\frac{b_{i} - s^i}{\mu }\Big ) - s^i B_i/\mu \right] <0. \end{aligned}$$

Thus, the reward function has a unique maximiser, and the model satisfies the first part of Assumption 1. Let us denote this maximiser as $s^{i*}$. Then

91 $$\begin{aligned} B_i + \exp \Big (\frac{b_{i} - s^{i*}}{\mu }\Big ) - s^{i*} B_i/\mu = 0. \end{aligned}$$

Since exponents are positive, we note that the above implies

92 $$\begin{aligned} s^{i*}/\mu > 1. \end{aligned}$$

We take a partial derivative of Eq. 91 with respect to $s^j$, where $j\ne i$, treating $s^{i*}$ as a function of $s^j$. Then

93 $$\begin{aligned} -\frac{1}{\mu }\exp \Big (\frac{b_{j} - s^{j}}{\mu }\Big ) - \frac{1}{\mu }\exp \Big (\frac{b_{i} - s^{i*}}{\mu }\Big )\frac{\partial s^{i*}}{\partial s^j} - \frac{1}{\mu }B_i \frac{\partial s^{i*}}{\partial s^j} + \frac{1}{\mu ^2} s^{i*} \exp \Big (\frac{b_{j} - s^{j}}{\mu }\Big ) = 0 \end{aligned}$$

which gives

94 $$\begin{aligned} \frac{\partial s^{i*}}{\partial s^j} = \frac{\exp \Big (\frac{b_{j} - s^{j}}{\mu }\Big ) \Big (\frac{s^{i*}}{\mu } - 1 \Big )}{\exp \Big (\frac{b_{i} - s^{i*}}{\mu }\Big ) + B_i} = \frac{\exp \Big (\frac{b_{j} - s^{j}}{\mu }\Big ) \Big (\frac{s^{i*}}{\mu } - 1 \Big )}{A}. \end{aligned}$$

From 92, we conclude

95 $$\begin{aligned} \frac{\partial s^{i*}}{\partial s^j} >0 \end{aligned}$$

and thus the model satisfies second part of Assumption 1. Now, we sum the partial derivatives over $j \in \{1,2,3,...,i-1,i+1,...,N\}$. We get

96 $$\begin{aligned} \sum _{j\ne i}^N \frac{\partial s^{i*}}{\partial s^j} = \frac{ \Big (\frac{s^{i*}}{\mu } - 1 \Big ) \sum _{j\ne i}^N\exp \Big (\frac{b_{j} - s^{j}}{\mu }\Big ) }{A}. \end{aligned}$$

Now, we note that

97 $$\begin{aligned} \sum _{j\ne i}^N\exp \Big (\frac{b_{j} - s^{j}}{\mu }\Big ) < B_i \end{aligned}$$

and thus

98 $$\begin{aligned} \sum _{j\ne i}^N \frac{\partial s^{i*}}{\partial s^j} < \frac{ \Big (\frac{s^{i*}}{\mu } - 1 \Big ) B_i }{A}. \end{aligned}$$

Furthermore, from Eq. 91

99 $$\begin{aligned} \Big (\frac{s^{i*}}{\mu } - 1 \Big ) B_i = \exp \Big (\frac{b_{i} - s^{i*}}{\mu }\Big ) \end{aligned}$$

which gives

100 $$\begin{aligned} \sum _{j\ne i}^N \frac{\partial s^{i*}}{\partial s^j} < \frac{ \exp \Big (\frac{b_{i} - s^{i*}}{\mu }\Big )}{A}. \end{aligned}$$

Now, we note that the maximiser has to be non-negative thus:

101 $$\begin{aligned} \exp \Big (\frac{b_{i} - s^{i*}}{\mu }\Big ) \in \Big (0, \exp \Big (\frac{b_{i}}{\mu }\Big )\Big ] \end{aligned}$$

Moreover, by definition

102 $$\begin{aligned} A = \exp \Big (\frac{b_{i} - s^{i*}}{\mu }\Big ) + \sum _{j\ne i}^N\exp \Big (\frac{b_{j} - s^{j}}{\mu }\Big ) + \exp \Big (\frac{a_0}{\mu }\Big ). \end{aligned}$$

Therefore,

103 $$\begin{aligned} \sum _{j\ne i}^N \frac{\partial s^{i*}}{\partial s^j}< \frac{ \exp \Big (\frac{b_{i} - s^{i*}}{\mu }\Big )}{A}< \frac{ \exp \Big (\frac{b_{i} }{\mu }\Big )}{\exp \Big (\frac{b_{i} }{\mu }\Big ) + \exp \Big (\frac{a_0}{\mu }\Big )} < 1. \end{aligned}$$

Consequently, the model satisfies the third part of Assumption 1. This concludes the proof. $\square$

## Appendix D: Motivation for Assumption 1-iii)

Let’s assume that other assumptions, 1(i) and 1(ii) hold. Let’s consider the perspective of the *i*-th agent and initially fix other agents’ margins $S^{(-i)}$ as well as the reservation margin $S^C$. Slightly abusing the notation, we will treat the reservation margin $S^C$ as the argument for the expected reward and the maximiser function *B*. The expected reward for the agent $r(S^1,S^2...,S^C)$ can be written as:

104 $$\begin{aligned} r(S^1,S^2...,S^C) := {\mathbb {E}}[R^i|S^1,S^2,..,S^N,S^C] = S^i f(S^i-S^1, S^i-S^2, S^i-S^3, ..., S^i-S^C), \end{aligned}$$

where *f* is the probability of offer acceptance

105 $$\begin{aligned} \begin{aligned}&f(S^i-S^1, S^i-S^2, S^i-S^3, ..., S^i-S^C) \\&\quad := \int _{\mathbb {R}} \Phi \left( -\frac{S^{i} - S^{C} +\omega \sqrt{1-\rho }x }{\sqrt{\tau ^2 + \rho \omega ^2}}\right) \prod _{j\ne i} \Phi \left( -\frac{S^i - S^{j} +\omega \sqrt{1-\rho }x}{\omega \sqrt{1-\rho }}\right) \varphi (x)dx. \end{aligned} \end{aligned}$$

Now, given the other assumptions, there is a maximiser $S^i_e$, such that

106 $$\begin{aligned} \begin{aligned} \frac{\partial r(S^1,S^2...,S^C)}{\partial S^i}(S^i_e)&= f(S^i_e-S^1, S^i_e-S^2, ..., S^i_e-S^C) \\&\quad + S^i_e f'(S^i_e-S^1, S^i_e-S^2, ..., S^i_e-S^C)= 0. \end{aligned} \end{aligned}$$

Now, let us increase all margins, including the reservation margin $S^C$ by some $\delta$. Then, the derivative at $S^i+\delta$ is:

107 $$\begin{aligned} \begin{aligned}&\frac{\partial r(S^1,S^2...,S^C)}{\partial S^i}(S^i+\delta ) \\&\quad = f(S^i+\delta -S^1-\delta , S^i+\delta -S^2-\delta , ..., S^i+\delta -S^C-\delta ) \\&\quad \quad + (S^i+\delta ) f'(S^i+\delta -S^1-\delta , S^i+\delta -S^2-\delta , ..., S^i+\delta -S^C-\delta )\\&\quad = f(S^i-S^1, S^i-S^2, ..., S^i-S^C) \\&\qquad + (S^i+\delta ) f'(S^i-S^1, S^i-S^2, ..., S^i-S^C). \end{aligned} \end{aligned}$$

Using equality from (106), we get:

108 $$\begin{aligned} \begin{aligned}&\frac{\partial r(S^1,S^2...,S^C)}{\partial S^i}(S^i+\delta ) = \delta f'(S^i-S^2, S^i-S^3, ..., S^i-S^C). \end{aligned} \end{aligned}$$

The probability *f* is a decreasing function thus if $\delta$ is positive (margins increased) the derivative of the reward function is negative. If $\delta$ is negative (margins decreased), the derivative of the reward is positive. Assuming that there is a single maximiser and assuming that the maximiser is monotonically increasing with respect to the reservation margin $S^C$ (this is a small extension of Assumption 1(ii)), we can conclude that the new maximiser lies between $S^i$ and $S^i+\delta$. Let’s take positive $\delta >0$. For the new maximiser $B_i(S^{(-i)}+\delta , S^C+\delta )$, we can write

109 $$\begin{aligned} B_i(S^{(-i)}+\delta , S^C+\delta ) \in [S^i, S^i+\delta ]. \end{aligned}$$

that is

110 $$\begin{aligned} B_i(S^{(-i)}+\delta , S^C+\delta ) \in [B_i(S^{(-i)}, S^C), B_i(S^{(-i)},S^C)+\delta ]. \end{aligned}$$

Now, from the assumed differentiability of the maximiser *B*, we can write first-order approximation for the maximiser given that all the margins changed by small $\delta$:

111 $$\begin{aligned} B_i(S^{(-i)}+\delta , S^C+\delta ) = B_i(S^{(-i)}, S^C) +\sum _{j\ne 1}\frac{\partial B_i}{\partial S^j} \delta + \frac{\partial B_i}{\partial S^C} \delta + O(\delta ^2). \end{aligned}$$

Since the maximiser lies between $B_i(S^{(-i)}, S^C)$ and $B_i(S^{(-i)}, S^C)+\delta$, we get:

112 $$\begin{aligned} B_i(S^{(-i)}, S^C) \le B_i(S^{(-i)}, S^C) +\sum _{j\ne 1}\frac{\partial B_i}{\partial S^j} \delta + \frac{\partial B_i}{\partial S^C} \delta + O(\delta ^2) \le B_i(S^{(-i)}, S^C)+\delta . \end{aligned}$$

Cancelling the $B_i(S^{(-i)}, S^C)$ from all sides, dividing by $\delta$ and taking $\delta \rightarrow 0$, we get the inequality:

113 $$\begin{aligned} 0 \le \sum _{j\ne 1}\frac{\partial B_i}{\partial S^j} + \frac{\partial B_i}{\partial S^C} \le 1. \end{aligned}$$

Consequently, given monotonicity of Assumption 1(ii), extended to include the reservation margin $S^C$, we can concur that the statement from Assumption 1(iii) holds for $\beta =1$.

To complete the proof of Assumption 1(iii), we need to establish that there exists $\beta$ smaller than 1 or equivalently that

114 $$\begin{aligned} \frac{\partial B_i}{\partial S^C} \end{aligned}$$

is bounded below by some positive quantity. This has not been done and would have to be assumed for the purpose of this paper.

The derivation above strongly implies that Assumption 1-iii) holds. Given Assumptions 1-i) and 1-ii), the only manner in which Assumption 1-iii) could be relaxed is if we allow $\beta$ to be equal to 1. In that case, we would no longer obtain the contraction due to best response play. Therefore, we would not have uniqueness of the Nash equilibrium, and there could in theory exist multiple, possibly mixed, Nash equilibria.

## Appendix E: Assumption Tests

The Assumption 1 were tested for a sample symmetric three-player setup. The environment was set up with the following parameters: $\omega =0.15$, $\rho =0.5$, $\tau =0.13$, and $S^C=1.$. Table 14 presents the partial derivative $\frac{\partial B_1}{\partial S^{2}}$. Table 15 presents the sum of the partial derivatives $\frac{\partial B_1}{\partial S^{2}} + \frac{\partial B_1}{\partial S^{3}}$. The numbers are subject to computational errors - evaluation of the derivative included application of finite difference scheme and Monte Carlo integration. Thus, the numbers are given with an accuracy of around 0.01. The calculations agree with the assumptions. The partial derivatives are non-negative and smaller than 1. While Assumption 1 requires that the derivatives are strictly positive, due to the approximation error, it is not possible to show that some of the presented derivatives are strictly greater than 0. The table shows that the calculations do not contradict any of the assumptions. Therefore, we can conclude that the proposed assumptions about the reward function are reasonable.

**Table 14:** Derivatives of $B_1(S^{-1})$ with respect to $S^2$ at $(S^2, S^3)$ points

$S^3$	$S^2$								
	0.10	0.20	0.30	0.40	0.50	0.60	0.70	0.80	0.90
0.10	0.19	0.15	0.08	0.03	0.01	0.00	0.00	0.00	0.00
0.20	0.27	0.26	0.18	0.08	0.02	0.00	0.00	0.00	0.00
0.30	0.35	0.37	0.31	0.20	0.07	0.01	0.00	0.00	0.00
0.40	0.41	0.49	0.46	0.36	0.20	0.07	0.00	0.00	0.00
0.50	0.43	0.54	0.59	0.53	0.39	0.20	0.06	0.01	0.00
0.60	0.43	0.55	0.65	0.67	0.59	0.41	0.20	0.05	0.01
0.70	0.43	0.55	0.65	0.72	0.73	0.63	0.42	0.18	0.05
0.80	0.43	0.55	0.65	0.73	0.78	0.77	0.64	0.40	0.16
0.90	0.43	0.55	0.65	0.73	0.78	0.81	0.77	0.61	0.35

**Table 15:** Sums of directional derivatives at $(S^2, S^3)$ points

$S^3$	$S^2$								
	0.10	0.20	0.30	0.40	0.50	0.60	0.70	0.80	0.90
0.10	0.38	0.42	0.43	0.44	0.44	0.43	0.43	0.43	0.43
0.20	0.42	0.52	0.55	0.57	0.56	0.55	0.55	0.55	0.55
0.30	0.43	0.55	0.62	0.66	0.66	0.66	0.65	0.65	0.65
0.40	0.44	0.57	0.66	0.72	0.73	0.74	0.72	0.73	0.73
0.50	0.44	0.56	0.66	0.73	0.78	0.79	0.79	0.79	0.78
0.60	0.43	0.55	0.66	0.74	0.79	0.82	0.83	0.82	0.82
0.70	0.43	0.55	0.65	0.72	0.79	0.83	0.84	0.82	0.82
0.80	0.43	0.55	0.65	0.73	0.79	0.82	0.82	0.80	0.77
0.90	0.43	0.55	0.65	0.73	0.78	0.82	0.82	0.77	0.70

## Appendix F: Model Posterior Updates

### F.1. Stationary Classical Model

The classical model assumes that for each of *N* available actions $\{a^1,a^2,...,a^N\}$ there is a stationary model parameter $\{\theta ^1,\theta ^2,...,\theta ^N\}$. The observation is the reward. After choosing *i*-th action at time step *t*, the reward/observation for the agent is sampled from a normal distribution with mean $\theta ^i$ and variance $\delta$, where $\delta$ is fixed and assumed within the prior information.

115 $$\begin{aligned} r_t\ |\ a_t\!=\!a^i \sim {\mathcal {N}}(\theta ^i, \delta ). \end{aligned}$$

The model is initialised with a prior on each of the parameters and the noise parameter $\delta >0$. Each parameter has a normal uncorrelated prior

116 $$\begin{aligned} \theta _i \sim {\mathcal {N}}(m^i_0, d^i_0), \end{aligned}$$

where for each *i*

117 $$\begin{aligned} m^i_0 =0 \qquad \qquad (d^i_0)^{-1} = 0. \end{aligned}$$

We will prove the formula for the posterior update using induction. At time $t=0$ the prior for each parameter is normal, as dictated by our setup.

Now, let us take the time step $t > 1$ and assume, without loss of generality, that action *i* was taken. For every $j\ne i$, the posterior for parameter $\theta _j$ is unchanged giving for every $j \ne i$

118 $$\begin{aligned} \theta _i \sim {\mathcal {N}}(m^i_t, d^i_t) \end{aligned}$$

with

119 $$\begin{aligned} m^j_t =&m^j_{t-1}, \end{aligned}$$

120 $$\begin{aligned} d^j_t =&d^j_{t-1}. \end{aligned}$$

To calculate the posterior of the parameter *i*, we will apply the Bayes rule (Jaynes, 2003) to the posterior probability of the observation of the reward $r_t$ given the parameter value $\theta _i$. We can write for the log posterior of the reward $r_t$

121 $$\begin{aligned} \begin{aligned}&\log p(r_t | \theta _i, m^i_{t-1}, d^i_{t-1} ) + \log p(\theta _i | m^i_{t-1}, d^i_{t-1} ) \\&\quad =\log p(\theta _i | r_t, m^i_{t-1}, d^i_{t-1} ) + \log p (r_t | m^i_{t-1}, d^i_{t-1} ), \end{aligned} \end{aligned}$$

where *p* denotes a probability function. In the above, we follow the notation from Jaynes (2003) and interpret the probability function $p(r_t|\theta _i, m^i_{t-1}, d^i_{t-1})$ as the probability that the reward at time *t* is equal to $r_t$ conditional on the *i*-th model parameter being equal to $\theta _i$ and the current posterior parameters being equal to $m^i_{t-1}, d^i_{t-1}$.

The last log term is constant with respect to $\theta _i$ and thus will be included in the normalisation constant. We note that

122 $$\begin{aligned} \log p(r_t | \theta _i, m^i_{t-1}, d^i_{t-1} ) = -\frac{1}{2}(r_t-\theta _i)\delta ^{-1} (r_t-\theta _i) + \text {const} \end{aligned}$$

and

123 $$\begin{aligned} \log p(\theta _i | m^i_{t-1}, d^i_{t-1} ) = -\frac{1}{2}(\theta _i - m^i_{t-1})(d^i_{t-1})^{-1} (\theta _i - m^i_{t-1}) + \text {const}. \end{aligned}$$

Thus

124 $$\begin{aligned} \begin{aligned}&\log p(\theta _i | r_t, m^i_{t-1}, d^i_{t-1} ) \\&\quad = -\frac{1}{2}(r_t-\theta _i)\delta ^{-1} (r_t-\theta _i) \\&\quad \quad -\frac{1}{2}(\theta _i - m^i_{t-1})(d^i_{t-1})^{-1} (\theta _i - m^i_{t-1}) + \text {const}. \end{aligned} \end{aligned}$$

Collecting the terms with $\theta _i$ and lumping constant terms, we get

125 $$\begin{aligned} \log p(\theta _i | r_t, m^i_{t-1}, d^i_{t-1} ) = -\frac{1}{2}(\theta _i - m^i_{t})(d^i_{t})^{-1} (\theta _i - m^i_{t}) + \text {const}, \end{aligned}$$

where

126 $$\begin{aligned} d^i_t&:= \frac{1}{\delta ^{-1} + (d^i_{t-1})^{-1}} = d^i_{t-1}\left( 1 - \frac{d^i_{t-1}}{d^i_{t-1} + \delta }\right) , \end{aligned}$$

127 $$\begin{aligned} m^i_t&:= d^i_t \left( \delta ^{-1}r_t + (d^i_{t-1})^{-1}m_{t-1}^i\right) . \end{aligned}$$

The new posterior is normal and the posterior parameters follow the update Equations (22) and (23). This concludes the derivation.

### F.2. Stationary Logistic Model

Let us reiterate the setup of the model. At each time step, the agent chooses a margin $s_t$ which translates to an action vector $a_t$ such that

128 $$\begin{aligned} a_t = [1, s_t]^T. \end{aligned}$$

The observation random variable $Y_t \in \{0,1\}$, the offer acceptance, is taken from a Bernoulli distribution, where the probability is assumed to follow a logistic function

129 $$\begin{aligned} Y_t \sim \text {Bernoulli}(p_t),\text { where }\quad p_t = \zeta \left( \theta ^T a_t\right) := \frac{1}{1+ \exp \left( -\theta ^T a_t\right) }, \end{aligned}$$

where $\theta \in {\mathbb {R}}^2$ is the model parameter vector. After sampling $Y_t$ to obtain $y_t$, the reward for the agent becomes

130 $$\begin{aligned} r_t = y_t s_t. \end{aligned}$$

The model is initialised with a prior normal distribution over the parameters. At time $t=0$

131 $$\begin{aligned} \theta \sim {\mathcal {N}}(m_0, d_0), \end{aligned}$$

where $d_0$ is necessarily large to encode the uncertainty about the exact value of the parameters. The prior density is denoted as $f_0$ and

132 $$\begin{aligned} f_0(\theta ) = \frac{1}{\sqrt{2\pi d_0}}\exp \left( -\frac{1}{2}\frac{(\theta -m_0)^2}{d_0}\right) . \end{aligned}$$

Let us denote the history of the actions up to time *t* as $a^{(t)}:= \{a_1,a_2,...,a_t\}$ and the history of observations as $y^{(t)}:=\{y_1, y_2,...,y_t\}$.

After *t* steps, we can write for the log posterior probability of the observations conditional on the action history, parameters $\theta$ and the prior

133 $$\begin{aligned} \log p(y^{(t)}| \theta , m_0, d_0) = \sum _{k=1}^t \log p(y_k | \theta ,m_0,d_0). \end{aligned}$$

In the above, we have conditioned the probability on the prior parameters $m_0$ and $d_0$ which only influence the probability of observations indirectly through the model parameters $\theta$. When conditioning on $\theta$, the information about $m_0$ and $d_0$ is redundant and can be omitted

134 $$\begin{aligned} {\mathbb {P}}(Y_k=y_k | \theta ,m_0,d_0) = {\mathbb {P}}(Y_k=y_k | \theta ). \end{aligned}$$

However, $m_0$ and $d_0$ will enter the posterior density on the parameters $\theta$ as the prior density $f_0$. Thus, for the purpose of mathematical rigour when applying the Bayes theorem, we will keep conditioning on $m_0$ and $d_0$.

The observations follow the Bernoulli distribution

135 $$\begin{aligned} {\mathbb {P}}(Y_k=1 | \theta ,m_0,d_0)&= \zeta (\theta ^T a_k) \end{aligned}$$

136 $$\begin{aligned} {\mathbb {P}}(Y_k=0 | \theta ,m_0,d_0)&= 1-\zeta (\theta ^T a_k) \end{aligned}$$

and thus we further expand the single log posterior term

137 $$\begin{aligned} \log {\mathbb {P}}(Y_k=y_k | \theta ,m_0,d_0) = y_k\log \left( \zeta (\theta ^T a_k)\right) + (1-y_k)\log \left( 1-\zeta (\theta ^T a_k) \right) . \end{aligned}$$

Using the Bayes rule, we can write for the posterior density of the model parameters

138 $$\begin{aligned}&p(\theta |Y_1=y_1,Y_2=y_2,....,Y_t=y_t, m_0,d_0)d\theta \nonumber \\&\quad \sim {\mathbb {P}}(Y_1=y_1, Y_2=y_2,...,Y_t=y_t | \theta , m_0, d_0) f_0(\theta )d\theta \end{aligned}$$

For the above to hold, we have to have

139 $$\begin{aligned}&p(\theta |Y_1=y_1,Y_2=y_2,....,Y_t=y_t, m_0,d_0) \nonumber \\&\quad \sim {\mathbb {P}}(Y_1=y_1, Y_2=y_2,...,Y_t=y_t | \theta , m_0, d_0) f_0(\theta ). \end{aligned}$$

Taking the logarithms of both sides, we get

140 $$\begin{aligned} \begin{aligned}&\log (p(\theta |Y_1=y_1,Y_2=y_2,....,Y_t=y_t,m_0,d_0)) \\&\quad = \log (f_0(\theta )) + \sum _{k=1}^{t}\Big [ y_{k} \log \left( \zeta (\theta ^T a_{k}) \right) + (1-y_{k}) \log \left( 1-\zeta (\theta ^T a_{k}) \right) \Big ] + \text {const} \\&\quad = \log (f_0(\theta )) + \sum _{k=1}^{t}\Bigg [y_{k} \log \left( \frac{\zeta (\theta ^T a_{k})}{1-\zeta (\theta ^T a_{k}) } \right) + \log \left( 1-\zeta (\theta ^T a_{k}) \right) \Bigg ] + \text {const} \\&\quad = \log (f_0(\theta )) + \sum _{k=1}^{t} y_{k} \theta ^T a_{k} + \sum _{k=1}^{t}\log \left( 1-\zeta (\theta ^T a_{k}) \right) + \text {const}. \end{aligned} \end{aligned}$$

At this point, to obtain a normal posterior, we use a second-order approximation for the second log term. We expand $\log (1-\zeta (\theta ^T a_{k}))$ around a mean *m* (yet to be specified)

141 $$\begin{aligned} \begin{aligned} \log (1-\zeta (\theta ^T a_{k}))&= -\log (1+\exp (\theta ^T a_{k})) \\&\approx -\log (1+\exp ( m^T a_k)) - \left( \frac{\exp (m^T a_k)}{1 + \exp ( m^T a_k)} \right) a_k^T(\theta - m) \\&\quad - \frac{1}{2} \left( \frac{\exp ( m^T a_k)}{(1 + \exp ( m^T a_k))^2} \right) (\theta - m)^T a_k a_k^T (\theta - m)\\&= -\log (1+\exp (m^T a_k)) - \zeta (m^T a_k) a_k^T(\theta - m) \\&\quad - \frac{1}{2} \zeta ( m^T a_k) \left( 1 - \zeta ( m^T a_k)\right) (\theta - m)^T a_k a_k^T(\theta - m). \end{aligned} \end{aligned}$$

Therefore, we can estimate the posterior for $\theta$ at time *t* as ${\mathcal {N}}(m_{t}, d_{t})$ with

142 $$\begin{aligned} d_{t}&= \left( d_0^{-1} + S_t \right) ^{-1} \end{aligned}$$

143 $$\begin{aligned} m_{t}&= d_{t+1} \left( d_0^{-1} \mu _0 + S_t m + \sum _{k=0}^{t-1}(y_{t-k} - \zeta ( m^T a_k)) a_k\right) \end{aligned}$$

144 $$\begin{aligned} \text {with} \quad S_t :&= \sum _{k=0}^{t-1} \zeta (m^T a_k)(1-\zeta ( m^T a_k)) a_ka_k^T. \end{aligned}$$

This concludes the derivation.
